# Assessing the contributions of an urban population health initiative to shift political priority towards cardiovascular health: three case studies from Brazil, Mongolia and Senegal

**DOI:** 10.1186/s12913-023-10432-8

**Published:** 2024-01-04

**Authors:** Jasmina Saric, Ann Aerts, Malick Anne, Joseph Barboza, Johannes Boch, Naranjargal Dashdorj, Diana Vaca McGhie, Adela Santana, Jason T. Shellaby, Suely Miya Shiraishi Rollemberg, Mariana Silveira, Peter Steinmann, Daniel Cobos

**Affiliations:** 1 Swiss Tropical and Public Health Institute, 4123 Allschwil, Switzerland; 2 University of Basel, 4003 Basel, Switzerland; 3 Novartis Foundation, Basel, Switzerland; 4 Division de la Lutte contre les Maladies non transmissible Ministère de la Santé et de l’Action Sociale, Dakar, Sénégal; 5 IntraHealth, Dakar-Fann, Dakar, Sénégal; 6 Onom Foundation, Ulaanbaatar, Mongolia; 7 American Heart Association, Dallas, Texas USA; 8 Secretaria Municipal da Saúde, Saúde, SP Brazil; 9 Instituto Tellus, São Paulo, SP Brazil

**Keywords:** Cardiovascular diseases, Kingdon framework, Urban population health initiative, Hypertension, Policy

## Abstract

**Background:**

The urban population health initiative was designed as a multidisciplinary, multisector programme to address cardiovascular (CV) disease, specifically hypertension and its underlying causes in the cities of Ulaanbaatar, Mongolia; Dakar, Senegal; and São Paulo, Brazil. This article aims to provide an overview of the history and dynamics of CV disease policy making in the three countries, to present the policy reform contributions of the initiative and its role in the policy agenda-setting framework/process in each country and to identify the enablers and challenges to the initiative for doing so.

**Methods:**

A qualitative case study was conducted for each setting from November 2020 to January 2021, comprised of a document review, semi-structured in-depth interviews and unstructured interviews with stakeholders involved in the initiative. The literature review included documents from the initiative and the peer-reviewed and grey literature with a total of 188 documents screened. Interviews were conducted with 21 stakeholders. Data collection and thematic analysis was guided by (i) the Kingdon multiple streams conceptual framework with the main themes being CV disease problems, policy, politics and the role of policy entrepreneurs; and (ii) the study question inquiring on the role of the urban population health initiative at the CV disease policy level and enabling and challenging factors to advancing CV disease policy. Data were thematically analysed using the Framework Method.

**Results:**

Each setting was characterized by a high hypertension and CV disease burden combined with an aware and proactive political environment. Policy outcomes attributed to the initiative were updating the guidelines and/or algorithms of care for hypertension and including revised physical and nutritional education in school curricula, in each city. Overall, the urban health initiative’s effects in the policy arena, were most prominent in Mongolia and Senegal, where the team effectively acted as policy entrepreneur, promoting the solutions/policies in alignment with the most pressing local problems and in strong involvement with the political actors. The initiative was also involved in improving access to CV disease drugs at primary health levels. Its success was influenced by the local governance structures, the proximity of the initiative to the policy makers and the local needs. In Brazil, needs were expressed predominantly in the clinical practice.

**Conclusions:**

This multi-country experience shows that, although the policy and political environment plays its role in shaping initiatives, often the local priority needs are the driving force behind wider change.

**Supplementary Information:**

The online version contains supplementary material available at 10.1186/s12913-023-10432-8.

## Background

Extraordinary improvements have been made over the last decades to the health of human populations, increasing both life expectancy and quality of life [1, 2]. Some of the most notable successes have been achieved in maternal, newborn and child health and in the control of infectious diseases. Yet, non-communicable diseases (NCDs) are ubiquitous as never before, and now especially hit low- and middle-income countries (LMICs). Their rise has led to increased global and national policy efforts to prioritize and address NCDs through a range of interventions that can be categorized into i) public health policy operating outside the health system to modify risk factors for NCDs (e.g. tobacco and alcohol regulations); ii) population screening policies; iii) healthcare provision policies; and iv) policies that strengthen the health system at a structural level [3–8]. However, policy-makers in LMICs face unique challenges to advancing solutions, especially in the area of healthcare delivery. Designed for delivering acute care, primary health care (PHC) systems in many countries do not have the capacity to address chronic patients’ needs. Policy makers are obliged to rethink PHC, to integrate chronic health services that cater to the need for long-term management and to coordinate care for chronically ill patients [9–12]. However, re-engineering health systems to include chronic health and care services faces multiple challenges, especially when resources are limited and needs are pressing at all levels. Change management within a health system to adapt processes, procedures, protocols and policies can also be a daunting task for health system managers in LMICs, as decades of donor investments into vertical, disease-oriented programmes have contributed to fragmented health systems. Resources for organising chronic NCD services have been inexistent or neglected, especially at PHC levels [13–15].

Besides PHC strengthening, the Declaration of Astana and the United Nations Political Declaration on Universal Health Coverage [16, 17] have repeatedly acknowledged that more efforts to prevent and control NCDs and their underlying determinants are essential to achieve Universal Health Coverage. Although the detrimental impact of smoking, alcohol abuse, physical inactivity and unhealthy food is widely proven, complex actions implying social, behavioural and economic policy solutions can only be realized through sustained political will and lasting actions. Partnerships that include public and private sector players can support such difficult changes by combining the public sector’s ability to prioritize and the private sector’s innovation power, to jointly increase health equity [17, 18]. Multisector collaborations have shown to be important drivers for whole-of-society approaches for tobacco control [19], healthy nutrition, physical activity [20, 21] and alcohol abuse prevention [22]. However, success requires high-level political commitment, a common understanding of the priority issues, and most importantly, a shared goal. It also requires good governance to orchestrate the mechanisms and processes through which different actors ‘articulate their interests, exercise their rights and obligations and mediate their differences [23].

As of 2020, 128 WHO Member States have developed a national integrated NCD policy, strategy and action plan that is at different stages of operationalization [23]. Despite this renewed global interest in NCDs [24] and investing into PHC, countries still struggle to implement meaningful changes for individuals and communities, often because of limited investment for national NCD plans. And while the 2020–2023 COVID-19 pandemic has put health at the top of national agendas, it has compromised national resources for health even more.

### Urban population health initiative

The urban population health initiative was designed as a multisector, multidisciplinary initiative to address hypertension, the prime risk factor for cardiovascular (CV) disease. It was set up to specifically address health system weaknesses such as inadequate healthcare pathways and medicines prescription practices and underlying social determinants of CV health, focussing on nutrition education. The initiative used a population health approach called CARDIO, shorthand for quality of **C**are, early **A**ccess to diagnosis and treatment, policy **R**eform, **D**ata and digital technology, **I**ntersectoral collaboration and local **O**wnership [25]. This international initiative was implemented in three cities – Ulaanbaatar in Mongolia, Dakar in Senegal and São Paulo in Brazil. It started in 2017 respectively 2018, depending on local approvals, and operated until 2019 in Ulaanbaatar and 2020 in Dakar and São Paulo. Over 1 to 2 years of implementation, blood pressure (BP) control rates tripled in São Paulo (from 12.3% to 31.2%) and Dakar (from 6.7% to 19.4%) and increased six-fold in Ulaanbaatar (from 3.1% to 19.7%) [26]. Although the three cities share a dense population, their size is quite different with Dakar and Ulaanbaatar housing a population of 1–1.5 million, as compared to a population of 12 million in São Paulo. They also display large socio-economic, cultural and health differences, with diverse health systems and governances. As such, this cross-continental, urban health initiative offers an opportunity to compare the impact of its CARDIO approach across different contexts.

Against this background, the purpose of our study was to i) describe the history and dynamics of CV disease policy making in Brazil, Mongolia and Senegal; ii) to present the “policy reform” contributions of the CARDIO approach and assess the role of the coalition in the policy agenda-setting framework/process in each country; and iii) to identify the enablers and challenges to the initiative.

## Methods

### Study design

A qualitative case study approach was used that included a document review, semi-structured in-depth interviews with key informants and unstructured interviews with stakeholders involved in the urban population health initiatives of the three cities − Ulaanbaatar, Dakar and São Paulo [27, 28]. The analysis was guided by Kingdon’s three streams framework – also referred to as multiple streams framework [27].

### Conceptual framework

**Fig. 1:**
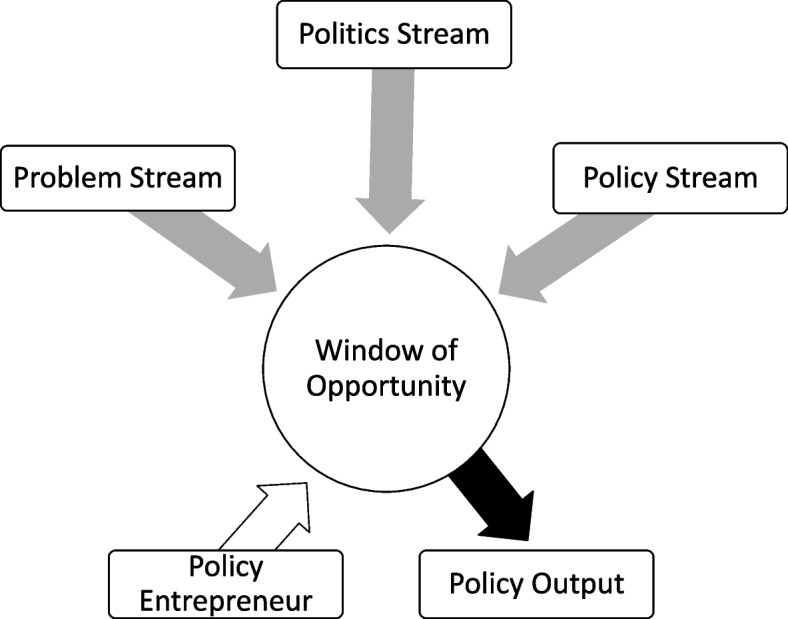
Components and concept of the Kingdon framework. Adapted from Sabatier 2007 [28]

An analysis of the NCD policy environment in Brazil, Mongolia and Senegal, before and during the implementation of the urban health initiative, was guided by Kingdon’s three streams framework [27]. In brief, the framework describes the policy-setting process as a dynamic of three streams: problems, policies and politics [27–29]. The problem stream involves recognizing issues as public concerns demanding government action, shaped by past government responses. Mechanisms making circumstances problematic include shifts in indicators, focusing events, or feedback, such as reports or evaluations. The policy stream involves continuous analyses of issues and their suggested solutions, coupled with discussions about these problems and potential responses. The politics stream encompasses events, such as shifts in national politics, government changes and advocacy campaigns by interest groups. In essence, for a policy to gain visibility on the agenda, it requires the recognition of a problem, a scalable solution, a supportive political environment and stakeholder backing. According to Kingdon, public policies emerge when policy entrepreneurs strategically connect a problem stream with a politics stream, utilizing windows of opportunity. This convergence, initiated by a policy entrepreneur, is facilitated when favourable circumstances align across the problem, policy and politics streams simultaneously. Policy entrepreneurs can emerge from any of these streams, depending on the situation, and they can be corporate or individual actors and advocates for proposals that can support the policy reform process (Fig. 1).

Kingdon-based case studies have been previously combined with the Framework Method to support data analysis [30, 31]. The Framework Method facilitates comparative techniques by reviewing data across a matrix rather than generating social theory. The method is often used to thematically analyse the transcripts derived from semi-structured interviews and it is deemed a flexible and systematic approach. For the current analysis assessing the role of the urban population health initiative within the local and national CV disease policy agenda-setting process, a combined approach of Kingdon three streams framework and the Framework Method was used.

### Document review

A document review was conducted to collect data that would support the understanding of the history and dynamics of CV disease policy making in Brazil, Mongolia and Senegal. First, all written documentation of the initiative was made available to the independent review team by the global coordinator of the initiative and the local implementation partners. Those documents included meeting minutes, monitoring reports, progress updates, policy notes and all peer-reviewed articles cited or published by the initiative (please see results section of each case study for further details). A total of 30 documents were screened by the lead author for the urban population health initiative for Brazil, 37 for Senegal, 36 for Mongolia and 12 cross-initiative documents.

Second, English-language peer-reviewed and grey literature was consulted to fill knowledge gaps using Web of Science, Google Scholar and websites of multilateral organizations (e.g. United Nations, World Health Organization). Non-initiative documents were additionally identified to fill in any gaps based on a cross-comparison of the three country settings. A total of 73 additional documents were screened in this process, by the lead author. The search to retrieve such information would generally be a combination of keywords including “CV disease”, one of the Kingdon streams, the setting and/or a keyword for the respective gap to be filled.

Relevant information was marked in each document in a first round of revision; in a second round of revision, the marked content was revisited in order to be discarded or extracted into a data sheet sectioned into the different themes/codes in the form of a matrix: i) problems, policies, politics and multilateral activities or financing, related to i) NCD/CV disease at local and national level; as well as ii) perceived initiative’s contributions at the policy level and its role in the policy agenda-setting process, along with iii) enabling and challenging factors to advancing NCD/CV disease policy. Information on strategies and solutions addressing NCDs as a disease group (i.e., CV disease, cancer, diabetes and chronic respiratory diseases) was included in the analysis, while information unrelated to CV disease and specific to cancer, diabetes, chronic respiratory diseases or other NCDs, was excluded.

### Interviews

Semi-structured interviews with key stakeholders were conducted to corroborate the findings from the document review. Interviewees were selected by purposive sampling, had to have collaborated with the urban population health initiative and have knowledge on at least one of the three Kingdon streams at national level. The initiative’s core group of stakeholders and partners was consulted, being the global coordinating organization, the local implementation partners and the local evaluation partners. In addition, government and external partners were interviewed. The number of interviews (21) depended on information saturation, i.e., the point when new data did not add any new information or a better understanding of the role of the urban population health initiative at the policy level in each case study, but rather confirmed what was previously expressed. When this point was reached, was estimated by the lead author during the data transcription and analysis. However, perspectives from a minimum of three types of stakeholders were sought in each setting to avoid introducing a predominant point of view. The interview guide featured first, the same main themes as were used for the literature review data extraction, namely: i) problems, policies, politics and multilateral activities or financing, related to NCD/CV disease at local and national level; as well as ii) perceived initiative contributions at the policy level and the role of the initiative in the policy agenda-setting process, along with iii) enabling and challenging factors to advancing NCD/CV disease policy. Second, and depending on the stakeholder, the interview guide contained questions that were added in order to fill gaps that arose from the document review and/or to deepen the understanding of processes and activities identified in the document review. Finally, for interviews with the global coordinator and the global coordination partners, the questionnaire featured comparative questions between the case studies/settings at the level of the three streams and the impact of the urban population health initiative (e.g., where was the politics stream most enabling or where did the initiative succeed the most in influencing the policy level). All semi-structured interviews were held by Zoom or Microsoft Teams between November 2020 and January 2021. Prior to that, unstructured interviews with initiative managers were conducted during site visits in September and October 2019. Interviews were conducted after verbal informed consent and lasted between 30 and 60 minutes. The 21 interviews in total were all digitally recorded and transcribed using Otter.ai software for interviews in English or manually for those in French (Table 1).

**Table 1:** Key informants on cardiovascular disease policy change in Brazil, Mongolia and Senegal and their association with the urban population health initiative

Role in the urban population health initiative	# representatives interviewed		
	Brazil	Mongolia	Senegal
Global evaluation partner	1	1	1
Local co-funder	1	0	0
Advisor	2	0	0
Local evaluation partner	0	1	1
Government partner	1	0	1
Global coordinator	2	3	2
Implementation partner	1	2	1
**Total**	**8**	**7**	**6**

### Data analysis

A thematic analysis of documents and interview transcripts was conducted based on the Framework Method [31]. We manually extracted content relevant to the main themes into an Excel sheet, thereby applying deductive coding. The main themes were provided by the Kingdon framework: (i) NCD/CV policy; (ii) politics; (iii) problems; and (iv) policy entrepreneurs; and additional questions asked by the study team aiming to assess (v) the role of the urban population health initiative within the three streams; (vi) the influence of operational level activities of the initiative towards the three streams; and (vii) enabling and challenging factors to advancing NCD/CV disease policy. Data within each case study was summarized according to code. Data obtained by the document reviews and interviews for each of the three case studies were methodologically triangulated to enhance the confidence in the data where the same information was obtained by both methods and pointed towards a gap in our insight where there was a contradicting finding. The process was based on the manual comparison of different data within each code within each case study, by the lead author. With each theme/code in a column across the matrix, each row contained the transcript from the various sources, including the documents and interviews. The matrix organization of the themes then allowed the researcher to read over each theme with the relevant transcript from every source. Findings were then synthesized for each theme.

## Results

The results are presented as a sequence of the three case studies, guided by the Kingdon framework, in the order of where the urban population health initiative first started. Each case study is structured – according to the three Kingdon streams – into problem, policy and politics stream. The final sub-section in each case study presents the policy entrepreneurs identified in recent years and the role of the initiative in enabling policies and/or other actors in acting as drivers of policy. Based on the main objective of the study, of describing the impact of the urban population health initiative, its drivers and challenges, the three streams are presented in a condensed form in the main body of the article, with a more in-depth description in Annex 1.

### The three Kingdon streams in Mongolia

#### Problem stream

In 1992, Mongolia transformed from a single-party Communist state to a multiparty democracy, accompanied by a stepwise implementation of free market reforms and rapid urbanization. The resulting changes in lifestyle contributed to growing national rates of hypertension and its CV complications. The first NCD STEPS survey in 2006 reported a 28% prevalence of hypertension among Mongolians aged between 15 and 64 years, which was confirmed in two subsequent surveys in 2009 and in 2013. The 2013 STEPS also revealed that approximately 27% of the participants never had their BP measured and that 72% of the patients diagnosed with hypertension were not under treatment. Of the remaining 28%, 21% did not achieve BP control while 7% reported successful BP controlled under medication [32]. In the latest STEPS survey, 2019, hypertension prevalence was at 23.6%. These are some of the reasons why Mongolia has amongst the highest rates of haemorrhagic stroke globally [33].

#### Policy stream

The government of Mongolia responded to the rise of NCDs and hypertension by identifying NCDs as a population health priority in 2000 [4]. A first National Programme for NCD risk factors was implemented during 2006–2013 [4, 34]. In 2010, the country launched a National Strategy on Healthy Diet and Physical Activity (2010–2021), as well as a Health Education Strategy and a National strategy on Information Education and Communication to Promote Healthy Behaviours. In 2011, clinical guidelines for arterial hypertension were established, while 2012 saw the adoption of a food law to improve food quality and support healthy diets, as well as a National Programme on Healthy City, District, Workplace and Schools [4]. According to several sources, preventive measures in more recent years (i.e. 2017–2020) included the national introduction of student and teacher books and tools on ‘Nutritional Education for primary school grades 1–6’, coupled to an annual health check-up in schools and workplaces tailored to age and gender (Table 2). Interviewees further stated that policy responses to tobacco consumption were among the most prominent and powerful policy responses to hypertension [35]. Since 2010, the Mongolian authorities also made progress on guidance for salt reduction, leading to a 10 year national salt reduction strategy (2015–2025). At health system level, both the Ministry of Health (MOH) and the National Health Insurance Fund were able to increase annual budgets for PHC during the implementation period of the urban population health initiative (Table 2). The National Health Insurance Fund increased the number of subsidized antihypertensive medications from 12 to 24, for the first time including fixed dose combination drugs, and increased the average coverage for antihypertensives costs by 5%. Overall, funding for National Health Insurance Fund subsidies for antihypertensive drugs increased by 50% in 2019 and by another 60% in 2021. Several respondents also noted that revised guidelines and standard algorithms for the management of hypertension were approved and implemented in all primary health centres of Ulaanbaatar during the initiative. Lastly, in 2019, a Nutrition Education curriculum was introduced for 1–6 grade students, together with teacher tools at the national level and with an annual check-up tailored to age and gender.

**Table 2:** Summary of the Kingdon framework’s three streams describing problem, policy and politics surrounding cardiovascular disease in Mongolia and outcomes directly or indirectly attributed to the urban population health initiative (*bold/italic*)

Problem stream	Policy stream	Politics stream
1992 Adoption of a new constitution accompanied by stepwise integration of free-market reforms and rapid urbanization, leading to a significant increase in the population of Ulaanbaatar (accounting for up to 50% of the total Mongolian population) 1997–2020 Multiple failures by different multilateral efforts to establish an integrated health information system 2006 STEPS survey in Mongolians aged 15–64 • Hypertension prevalence of 28% • Diabetes prevalence of 8% (5% increase since 1999) • 91% of the surveyed population had ≥1 risk factor for developing NCDs and 21% had ≥3 risk factors 2009 STEPS survey • Hypertension control rates declined from 21% in 2006 to 12% in 2009 2011 First national baseline assessment on salt consumption • Average daily salt intake of 11.06 ± 5.99 g – more than double the WHO recommended 5 g daily 2013 STEPS survey • Hypertension prevalence persisted around 28% between 2006 and 2013 • Hypertension control rates declined from 21% in 2006, to 7% in 2013* • An estimated 27% of individuals never had their BP measured • Of individuals previously diagnosed with hypertension, 72% were not treated by medication, 21% were treated but failed to achieve BP control and 7% achieved control 2019 STEPS survey • The prevalence of hypertension is 23.6%	2000 Alcohol control law adopted and updated in 2009 2005 Tobacco control law adopted and updated in 2012 2006 Introduction of the National Programme on Integrated Prevention and Control of NCDs 2011 Clinical guidelines for arterial hypertension 2012 Adoption of a food law and National Programme on Healthy City, District, Workplace and Schools 2012–2013 Pinch Salt intervention, associated with a reduction of 2.8 g of salt intake 2015–2025 National salt reduction strategy aiming to reduce salt intake by 30% by 2025 ***2016–2018 NHIF increases financial support to primary health clinics by 306% and raises reimbursements for hypertension medications, financed in part by a nationwide tobacco tax.*** ***2016–2018 NHIF increases the number of subsidized antihypertensive medications from 12 to 24, including fixed combination drugs for the first time, and increases their average cost coverage by 5%.*** ***2017–2019 MOH increases annual direct budget for primary healthcare by 19%*** ***2018 New national hypertension guidelines and standard algorithm of care approved by MOH and implemented across all 142 primary health centres in Ulaanbaatar, including a lower hypertension diagnosis and control target (130/80 mmHg) and a lower age limit for screening (18 years)*** ***2019 ‘Nutrition Education 1–6 grade’ student, teacher tools developed for schools, introduced on national level together with an annual check-up tailored to age and gender.***	1997 Start of the World Bank & UN Development Programme collaboration with the Ministry of Finance on a 10-year poverty alleviation plan, investing and mobilizing over $470 million, including $20 M for eHealth software development with the MOH. 2007 US MCC programme and Mongolian Government sign a five-year contract including $42 million devoted to NCD (including a large scale hypertension initiative) 2010 WHO regional consultation on salt reduction in Singapore 2011–2016 Luxembourg Development Cooperation funds an €11-million project, to improve health services including for CV disease 2012–2016 Asian Development Bank funds a health sector development programme, providing loans totalling $29.9 million for hospital infrastructure, eight technical assistance operations and three grant projects for infrastructure, health policy development and other core clinical focus areas. 2017 Analysis by the UN Inter-Agency Taskforce demonstrating that, compared to interventions for tobacco control, alcohol abuse and CV disease healthcare services, limiting salt use offers highest return on investment ***2019 New Bloomberg ‘Resolve to Save Lives’ grant agreement with the Onom Foundation, aimed at scaling the CARDIO interventions nationwide.*** 2019 MOH with support of WPRO starts Mon-PEN /HEARTS pilot initiative to improve prevention and early screening of NCD in one district of Ulaanbaatar and one province 2021 MOH starts rolling out Mon-PEN/HEARTS initiative in an additional seven provinces.

*The 2013 numbers are disputed – see Annex 1; *CV*, cardiovascular; *MCC*, Millennium Challenge Corporation; *MOH*, Ministry of Health; *NCD*, Non-communicable disease; *NHIF*, National Health Insurance Fund; *PEN*, package of essential non-communicable (disease interventions); *UN*, United Nations; *WHO*, World Health Organization; *WPRO*, World Health Organization / Regional Office for the Western Pacific

#### Politics stream

At international level, several externally funded, government-led projects supported the local health system and its response to NCDs in Mongolia, illustrating a growing acknowledgement of their importance for the country’s development. Donors included the World Bank, the United Nations Development Programme and the Asian Development Bank. One of the earliest initiatives was the US funded five-year Millennium Challenge Corporation programme, which started in 2007. It invested $42 million in capacity building, behaviour change and health system strengthening for NCDs. The Millennium Challenge Corporation funded the 2009 and 2015 STEPS surveys and developed the first hypertension guidelines. Interviewees highlighted that, while the Corporation had been instrumental to bring national attention to NCDs, its effects on healthcare workers capacity building for hypertension and behaviour change at the PHC level were not sustained [36]. The implementing partner of the Millennium Challenge Corporation programme was Onom Foundation (same as for the urban health initiative), together with other NGOs. The following explanation was given for the lack of sustainability:

*Particularly when it comes to primary care, it is difficult to sustain that level [of training on hypertension guidelines]. This is because of the nature of how primary care is working; they [general practitioners] usually work in one place, for approximately 2 years, and then they change place and try to go to secondary or tertiary care, because primary care is the worst paid.” (Representative Onom Foundation).*

After completing the urban health initiative here described, the Onom Foundation was awarded a Bloomberg grant from ‘Resolve to Save Lives’, to scale the CARDIO approach interventions nationwide in 2019. The three streams are described in more detail in Annex 1.

#### Policy entrepreneurs and the role of the urban population health initiative

**Fig. 2:**
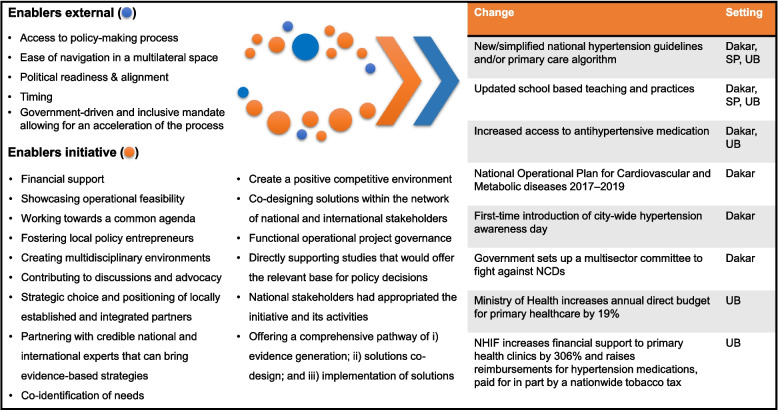
Enabling external factors (three streams) and potential urban population health initiative influence on positive policy and operational changes. NCD, non-communicable disease; NHIF, National Health Insurance Fund; SP, São Paulo; UB, Ulaanbaatar

After a decade of increasing awareness for hypertension, its associated risks and underlying determinants, the urban health initiative triggered renewed attention on improving urban CV population health. When it was launched, the mayor of Ulaanbaatar acknowledged that a new approach was urgently needed to reverse the high burden of stroke in the city. Strongly aligned with that local need, the initiative was able to directly and indirectly lead to additional CV disease policy and health systems improvements (Table 2). It demonstrated the feasibility of adequately managing hypertension at PHC level, improving health outcomes in a setting where the CV disease burden had traditionally been addressed at secondary and tertiary health facilities. The multisector initiative acted as a driver for improving CV population health in Mongolia, through creating eye-opening interactions between government stakeholders and multidisciplinary partners, backed by international expertise (Fig. 2). By showcasing the fitness of the PHC level management of hypertension and creating space for conversation, the core team of the initiative enabled a convergence of the three streams and thereby acted as policy entrepreneur for a number of policy-level changes (see policy and problems sections and Table 2).

Several interviewees reported that the strongest impact of this urban health initiative on policy and systems had been the revision of national hypertension guidelines with standardization of its management in PHC and the translation of this guidance into user friendly standard algorithms of care (Table 2).

A major driver of the success seemed to be a mutually reinforcing relationship between the urban population health initiative and its implementing partner in Mongolia – Onom Foundation. The initiative strengthened the influence of Onom Foundation at the policy level as described as follows:

*“Onom were able to build on top of the urban population health initiative and to benefit from that multidisciplinary initiative and positioning to drive change. The initiative and the advocacy around CV diseases and primary care gave them leverage to get closer to the policy makers.” (Representative Novartis Foundation).*

Given that Onom Foundation was an independent organization that was well represented at national level, it was instrumental in supporting the health policy reforms. For example, Onom Foundation was perceived as one of the main advocates for the change in PHC budgets and the increased coverage of antihypertensive medications (Table 2). In addition, their relevance to the city mayor, MOH and other policy makers, was perceived as further enhanced thanks to the organization’s role in the multisector initiative. Engaged on several fronts such as healthcare and schools, the Onom Foundation played an important role in driving the agenda and implementation of the Nutrition Education programme in schools. The key role of Onom Foundation has been described as follows:

*“They [Onom Foundation] were the hub in a wheel with many spokes; one spoke was the urban population health initiative, one spoke was their schools, one spoke was some of their own education and advocacy, but it created a greater circle of change.” (Representative Novartis Foundation).*

For the MOH to increase its direct PHC budget (Table 2), respondents identified several drivers and policy entrepreneurs, including the Mongolian Association for Family Health Centres and the Asian Development Bank that was aiming to improve health sector financing together with the Ministry of Finance. The Onom Foundation played an advisory and advocacy role in these budget increases.

Overall, the successful results of the CARDIO approach in Ulaanbaatar − a six-fold increase in BP control after 2 years of implementation − created a mind-set shift for local authorities to acknowledge the importance of operational experience for health planning and resource allocation to addressing the leading cause of death in the country. It also paved the way for new financial support, in the form of a grant from ‘Resolve to Save Lives’ to support the national rollout of the CARDIO approach.

### The three Kingdon streams in Senegal

#### Problem stream

Historically, public health priorities in Senegal were focusing on infectious diseases and mother and child health, while the country now faces a rapidly increasing burden of CV diseases. The size of the problem was insufficiently understood until the 2015 national STEPS survey [37] revealed hypertension to be prevalent in 30% of the adult population (18–69 years), (increasing from 15.3% in 18–29 year olds, to 64.2% of those aged 60–69). Less than half (46%) of the hypertension patients were aware of their condition, 17% reported taking antihypertensives and only 8% achieved BP control (unpublished data). In 2016, stroke was the fifth major cause of death in Senegal and an estimated 17% of all deaths were attributed to CV diseases.

#### Policy stream

At policy level, the National Health and Development Plan 2009–2018 (Plan National de Développement Sanitaire) commented on the expensive care for NCDs and the resulting burden on Senegalese households. Yet, prior to 2014, barely any relevant policies or strategies were in place to address that disease group [38]. When the NCD Division was created within the MOH, it included a CV disease unit that was tasked with developing a National Operational Plan for Cardiovascular and Metabolic Diseases 2017–2020 [39] (Table 3). The plan was based on a situational analysis outlining gaps and opportunities for detecting, managing and controlling cardiovascular and metabolic diseases. It specifically suggested to develop a standard algorithm for hypertension management, to improve quality of care at PHC level and to improve access to antihypertensives. This document, developed with support of the urban health initiative, framed the efforts to address CV diseases and their main risk factors. The subsequent 2018–2028 National Health and Social Development Plan also has a strong orientation towards NCDs. While Senegal introduced three tobacco intervention policies in 2014 [40], the NCD National Guidelines from 2016, were translated in 2017–18 into standard algorithms of care for hypertension at PHC level (Table 3), coupled to a new patient pathway for hypertension including a long term approach to coordinated care. The latter attributed extended responsibilities to nurses and community health workers at the frontline. Another major progress was booked with the improved access to antihypertensive medicines following the national drug list revision in 2018. It allowed antihypertensives such as amlodipin and captopril to be prescribed at lower level health facilities such as community health posts. In addition, the World Hypertension Day was celebrated for the first time in 2018, and the primary school curriculum was revised by 2020 at the city level, to include education on NCD risk factors.

**Table 3:** Summary of the Kingdon framework’s three streams describing problem, policy and politics surrounding cardiovascular disease in Senegal and outcomes directly or indirectly attributed to the urban population health initiative (*bold/italic*)

Problem stream	Policy stream	Politics stream
2015 STEPS survey • Hypertension prevalence estimated at 29.8% in 18–69 year olds (ranging from 15.3% in people aged 18–29 years, to 64.2% in the 60–69 year old group) • 46% of the hypertension patients are aware of their condition • 17% of the hypertension patients report taking antihypertensive medicines, while only 8% achieve BP control • Stroke considered the fifth major cause of death in Senegal 2015 National Agency of Statistics and Demography reports frequent shortages of anti-hypertensive medicines. E.g. enalapril available in only 3% of the health centres and health posts, thiazide diuretics in 1%, atenolol in 3% and amlodipine in 33% 2016 MOH establishes protocols for standardizing hypertension management, covering all levels of the health system 2018 WHO NCD report • NCDs estimated to account for 42% of all deaths in Senegal, with 17% of the deaths attributed to CV disease in 2016	2014: Adoption of Tobacco Control Act • By 2016, a comprehensive ban on all tobacco advertising, promotion and sponsorship was achieved • As of 2019, Senegal was the only LMIC offering full smoking cessation support services 2016: National Guidelines for six priority NCDs ***2017: National Operational Plan for Cardiovascular and Metabolic diseases 2017–2020*** ***2017–18: NCD National guidelines translated into a single standard algorithm of care for managing hypertension in PHC services.*** *** • Nurses of PHC centres and health posts now allowed to refer hypertension patients to 2nd or 3rd level of care (task shifting policy)*** *** • Every adult ≥ 18 presenting in a health facility is being screened for high BP.*** *** • National policy enabling community health workers to measure BP, refer patients for confirming hypertension diagnosis, and follow up for patients under treatment*** 2018: National Tobacco Control Strategic Plan 2018–2022 2018: National Health and Social Development Plan 2018–2028 ***2018: National essential drug list revised to allow prescription of antihypertensives such as amlodipin and captopril also at lower level health facilities (community health posts and health centres).***	1975: Belgium Development Cooperation starts supporting health sector in Senegal, focusing on the PHC and overall health system strengthening 1998: National Health and Development Plan 1998–2007 2008: National Health and Development Plan 2009–2018 2013: NCD Division created within MOH with a unit focusing on CV diseases 2014: ‘Emerging Senegal Plan’ 2014–2023 2016: Belgium Development Cooperation supports harmonization of guidelines for six priority NCDs 2017: National Operational Plan for Cardiovascular and Metabolic diseases 2017–2019 2018: Stakeholder workshop, chaired by the WHO local office, contributing to the strengthening of local guidelines and standard algorithms of care for hypertension 2018: National Health and Social Development Plan 2018–2028 ***2018/2019: World Hypertension Day celebrations in Dakar*** ***2020: Government sets up a multisector committee to address NCDs***

*BP* Blood pressure, *CV *Cardiovascular, *LMIC *low- and middle-income country, *MOH *Ministry of Health, *NCD *Non-communicable diseases, *PHC *Primary health care, *UN *United Nations, *WHO *World Health Organization

#### Politics stream

Momentum against NCDs was gained in Senegal following the 2011 United Nations high-level meeting on NCD prevention and control, and the 2015 STEPS survey documenting the size of the hypertension problem. The NCD Division at the MOH was created earlier, as NCDs were recognized as potential challenges to economic growth and development. Health was also deemed essential to achieve the strategy for an emerging Senegal by 2035 (Plan Sénégal Emergent; PSE). Several interviewees cited the Minister of Health at the time of the urban population health initiative’s launch as a key driver and advocate for establishing the NCD Division at the MOH and for the National Operational Plan, as follows:

*“She played an instrumental role here in the sense that she asked us to come in and help with this operational plan. She was really spearheading this effort from moving beyond the high level strategy documents that were reflecting global health policy recommendations towards ‘can we tangibly target this’, and that’s where we introduced this much bigger focus around primary care.” (Representative Novartis Foundation).*

At international level, the Belgian Development Cooperation was supporting PHC strengthening in Senegal for almost five decades. It spearheaded the harmonization of guidelines for six priority NCDs, including CV disease in partnership with the MOH and PATH, who was one of the implementation partners of this initiative too. In 2020, the MOH established a multisector committee to address NCDs, relating back to the National Health and Development Plan 2009–2018, which had specified that controlling NCDs required intra- and inter-sector collaboration, involving both private and public sectors. According to an MOH interviewee, the committee involved sport-, industry-, urban planning- and justice sectors. The urban population health initiative’s multisector partners enabled the involvement of the private sector (Table 3) [41]. The three streams are described in more detail in Annex 1.

#### Policy entrepreneurs and the role of the urban population health initiative

The urban population health initiative started in Dakar at a time point when NCDs were recognized as potential challenges to economic growth and development. Several interviewees stated that before starting its interventions, the initiative supported the MOH NCD Division in developing a first National Operational Plan for cardiovascular and metabolic diseases. In particular, it suggested to develop a standard algorithm for hypertension management, to improve quality of care at PHC level and to improve access to hypertensives, and therefore represented a baseline to which the initiative’s subsequent interventions were compared. The National Operational Plan suggested to develop a standard algorithm for hypertension management, to improve quality of care at PHC level and to improve access to antihypertensives. The co-development of this plan between the multisector partners and the MOH created a high degree of local ownership that embedded the urban health initiative within the national plan from the onset. Ensuring close and strategic alignment, supporting interventions on the ground, and engaging in discussions around priorities for policy change, appear to have created a window of opportunity that gave rise to several policy progresses (Fig. 2; Table 3).

The development of the national standard of care for hypertension and its translation into an algorithm for PHC workers and the revision of the essential drugs list, were perceived as being the most impactful interventions, with the initiative leveraging the NCD guidelines developed in 2016 by the Belgian Development Cooperation and PATH. This was the basis for developing the standard algorithm for hypertension, which included experts from the Senegalese cardiology society and the MOH NCD Division. Discussions on the benefits of task shifting of responsibilities between health professionals and less skilled health workers, and on the integration of the standardized hypertension management in PHC, resulted in more policy changes (Table 3). Sharing knowledge and evidence from settings where task shifting for hypertension management exists, facilitated the trainings and integration of BP measurement and follow up in the workflow of community health workers. The urban population health initiative empowered community health workers to demonstrate their ability to relieve workload from health professionals, and at the same time it gave the MOH Division for Community Health an argument to expand community health workers’ profiles.

*“The impact of the urban population health initiative is strong…The initiative started on the basis of a situational analysis and from this situational analysis we saw the gaps and the need to have protocols, which lead to the revision of these protocols and their adoption.” (Representative NCD Division, MOH).*

Being asked whether any operational activities of this imitative influenced policy making, the NCD Division’s representative further elaborated:

*“This is the most critical point of the initiative. We knew from the context of infectious diseases that there is an important role for community actors. Now, we have to change the paradigm to make community actors play a role in NCDs. For their involvement, I think the project played the pioneering role which enabled us to engage community actors more proactively in the fight against NCDs.”*

Several factors enabled a successful regulatory change to improve access to antihypertensive medicines. PATH, one of the implementing partners of the initiative, conducted a specific study in Dakar around the accessibility and availability of hypertension medicines; it showed that some of the drugs were only available at PHC and higher level facilities, but not at health posts. Given the widespread agreement on the standardized hypertension algorithm that stipulated the importance for patients to access their antihypertensive medicines at all levels of care, the initiative funded the hypertension study and supported the advocacy leading to this regulatory change. As this was already a high priority topic for the MOH and the National Drug Stores (Pharmacy National d’Approvisionnement), timing of this study was ideal given that its results were timely to inform the revision of the national drugs list in 2018.

Finally, several interviewees mentioned how it was the initiative’s work around workplace health [41] that kick-started a replication of similar activities in schools in Dakar, resulting in a revised primary school curriculum to include NCD risk factors. While the NCD Division had initiated discussions to update the school curriculum with NCD education prior to the urban health initiative, these had not advanced. The NCD Division eventually sought to revisit the curriculum revision supported by the initiative. Represented by PATH, the initiative convened both the MOH and Ministry of Education to jointly outlined a plan for the school curriculum revision, by involving experts in NCD and education and several consultation sessions in schools. This resulted in a collaboration between PATH and the NCD Division with the Schools’ Medical Control Division, to integrate NCD risk and healthy behaviour education in the student curriculum.

## The three Kingdon streams in Brazil

### Problem stream

An increasing sedentary lifestyle and changes in food habits increased the prevalence of obesity in Brazil from 11.8% in 2006 to 18.9% in 2016, according to the biannual national Vigitel survey on NCD risk factors. Average national salt consumption in Brazil is more than double that recommended by WHO, and NCDs are the leading cause of death, accounting for 74% of overall mortality and 28% of the CV mortality. Population-based surveys estimate the prevalence of hypertension in adults between 23% and 30% [42], with over 80% of those patients being aware of their diagnosis [42]. Access to hypertension medicines is reportedly high (~ 75%), yet studies suggest that between 35% and 55% of the patients treated by medication do not achieve BP control [42, 43]. According to several interviewees, a major challenge to managing hypertension and CV diseases in Brazil is the lack of a reliable estimate of national prevalences, the lack of inclusion of hypertension metrics in the national health information system, and the lack of a registry on CV diseases.

### Policy stream

The creation of the Brazilian Unified Healthcare System (Sistema Único de Saúde; SUS) in 1988 was a major achievement in terms of public health. More than 75% of the country’s population rely on the SUS for their health services. Significant efforts were made to strengthen the capacity and coverage of the primary health system to tackle NCDs, including hypertension. The 10-year Strategic Action Plan to Tackle NCDs (2011–2022) included the first goals for the MOH to develop public policies and actions to prevent and control NCDs. The plan guided Brazil’s states and municipalities in addressing NCDs and their underlying determinants – tobacco, unhealthy diet, physical inactivity and alcohol abuse [44]. Brazil introduced tobacco control laws as early as 1989 and became a widely cited best practice for managing to reduce smoking in its population from 35% in 1989 to 15% in 2021 [5, 45, 46]. Health and nutrition are incorporated in school curricula and standards for food served in schools and workplaces are established [44]. Mandatory food labelling was adopted in 2003 during the harmonization of Mercosur, taking effect in 2007 [6]. Between 2007 and 2011 policies for salt, sugar and trans-fat in processed foods were updated.

At the São Paulo municipal level, the urban population health initiative supported the provision of updated simplified and standardized hypertension guidelines and algorithms for service delivery in PHC, a national guidance for healthy school activities and hypertension “screening corners” in primary health centres. Those screening corners were introduced in the PHC centres of the city where BP is measured for everyone visiting the facility, regardless the reason (e.g. as a patient, caregiver or visitor) but also in other hotspots. The importance and the locations of those screening corners are further explained in detail by the following statement:

*“What makes the biggest impact is really the early detection, because people don’t know they are sitting on a problem like high blood pressure. So when you offer these diagnoses within the health system by obligatory screening corners in São Paolo, everybody who passes for whatever reason, also gets their risk factors assessed. We also introduced them [screening corners] outside of the brick and mortar of the health system,* e.g.*, in the pharmacies, schools, barber shops the bus stations. (Representative Novartis Foundation).*

This was recognized as an innovative way of addressing one of the main gaps in the hypertension chain in São Paulo (early detection) and was included into the WHO’s SDG good practices catalogue [47].

Finally, the NCD Technical Division within the Primary Care Coordination of the Health Department of the City of São Paulo has designed in 2022 the first municipal strategy to address NCDs as part of the Municipal Health Plan. The strategy outlines the objectives and annual targets for the management of NCDs by 2025 including the annual roadmap for 2022, focused on the consolidation of the NCD care line in the city. This strategy was strongly influenced by the urban population health initiative and stipulates the replication of core elements of the developed model and its care pathway across the entire municipality.

### Politics stream

According to several interviewees, the SUS truly delivered a structure for PHC that has been resilient to political change so far, thanks to its wide political consensus, and the health system now seems to be a driver for NCD control. At the municipal level, São Paulo state/municipality which hosts São Paulo city, the largest and economically most important city in Central- and South America, has given rise to major national NCD policies. First, the fight for full health care coverage for people with diabetes started in São Paulo state, according to sources, with approval in the early 2000s, subsequent scale-up throughout the country and implementation at the federal level. The same happened with smoking in public and private areas and closed places. Similarly, the political will for action against NCDs was strong in 2018 in São Paulo, as the city government was aiming to reduce waiting lists for specialist examinations by ramping up NCDs service capacity in the PHC system and by making short-term use of private hospital capacity (Corujão da Saúde) [48]. The three streams are described in more detail in Annex 1.

### Policy entrepreneurs and the role of the urban population health initiative

All interviewees agreed that the urban health initiative, locally knowns as *Cuidando de Todos*, aimed at changing policy and practice at the municipal rather than the federal level given the size and complexity of Brazil and its multiple governance levels (i.e. federal, state and municipal). In the Design Thinking workshop between the initiative’s partners and the local authorities in São Paulo to co-create the initiative’s interventions [49], improving detection and management of hypertension and PHC processes emerged as a priority, more so than policy reform (Fig. 2). By focussing on this operational need, the initiative’s impact was felt primarily on the level of health system process optimization. The initiative managed to demonstrate the beneficial effect of simplified standardized hypertension guidelines, of a national guidance for healthy school activities (Table 4) and of hypertension “screening corners” in primary health centres. Importantly, through its partnership and rigorous measurement system (i.e. progress monitoring and outcomes evaluation framework), the initiative was perceived to have facilitated securing funds from the Interamerican Development Bank for the city authorities, to strengthen primary care in São Paulo − as credited by the city’s Secretary of Health.

**Table 4:** Summary of the Kingdon framework’s three streams describing problem, policy and politics surrounding cardiovascular disease in Brazil and outcomes directly or indirectly attributed to the urban population health initiative (*bold/italic*)

Problem stream	Policy stream	Politics stream
2006–2020 Launch of the nationwide Vigitel surveys on NCD risk factors 2008 NCD Guidelines 2008 Brazilian Household Budget Survey 2008–2009 • Average national salt consumption in adults – is more than double (12 g per day) the WHO recommended 5 g per day • Salt and salt-based condiments added to meals represent 76% of daily salt intake 2014 First registry for hypertension and diabetes HIPERDIA discontinued 2016 National Vigitel Survey • Obesity prevalence increased from 11.8% in 2006 to 18.9% in 2016 • Between 1974 and 2009, overweight in adults increased from 18.5 to 50.1% for men and from 28.7 to 48% for women 2018 WHO NCD country profiles • NCDs account for 74% of all deaths in Brazil, and 28% are caused by CV disease s 2020 A comprehensive national estimate of hypertension prevalence is unavailable. However, city and/or state-based studies and surveys suggest: • A hypertension prevalence in adults of 23–30% Despite high access to medicines around 35 to 50% of hypertension patients treated by medication do not achieve BP control	2003 Brazil launches a national policy for the health of elderly persons, following the WHO approach for Active Aging 2003 Adoption of mandatory food labelling law (including trans-fats), taking effect in 2007 2003 Agreement between ABIA and MOH to reduce salt, sugar and trans-fat content in processed foods, renewed in 2010 2006 Approval of a broad health promotion policy including a series of intersectoral actions, health education, disease and risk factor monitoring, and health care promotion centred around healthy nutrition, physical activity, smoking cessation and reduction of the harmful use of alcohol. 2007 National policy for alcohol includes educational actions, advertisement and sale regulations, law enforcement for drinking and driving, and provision of care for those with alcohol-related problems 2009 A national legislation is enacted that requires at least 30% of the national school lunch budget to be spent on fresh foods from local agricultural production and family farms. 2010 Launch of 10-year Strategic Action Plan to Tackle NCDs 2011–2022 2014 Adoption of Tobacco Control Act By 2016, a comprehensive ban is introduced on all tobacco advertising, promotion and sponsorship, including at the point-of-sale	1988 Creation of the unified Brazilian Healthcare System 2003 WHO and the Food and Agriculture Organization recommend that ‘trans’ fat consumption should be less than 1% of the total daily energy calories 2003 Harmonization process within Mercosur to make food labelling mandatory 2003 FCTC holds the first international public health convention under the auspices of the WHO • Brazil acts as Vice-President of the Working Group that prepared the first draft of the convention • Brazil chairs the FCTC Intergovernmental Negotiation Body and heads the working group that prepared the first Conference of Parties 2005 To support local health promotion activities, the MOH transfers US$2 million to 27 state capitals. By 2009, the allocation had increased to US$25 million, distributed entirely to 1277 states and municipalities 2011 Agreement signed to establish goals for the reduction of salt in food as a priority 2014 Brazil appointed to preside the FCTC Secretariat

*ABIA* Brazilian Association of Food Industries, *FCTC *Framework Convention on Tobacco Control, *MOH *Ministry of Health

The standardized approach to hypertension management was also seen favourably, as it was developed together with the city authorities, professional medical associations and civil society organizations, and lead to a new best practice for policy making in health. Several operational processes were modified by the initiative, such as training health managers in evidence-based decision-making, promoting self-care strategies for patients and task shifting between healthcare providers. These operations were rolled out across the city, to the entire health corps through a combination of online continuous medical education, in person trainings and a digital clinical decision support system. The process started in 2018, when the initiative, in collaboration with the São Paulo Cardiology Society, started to simplify the existing Brazilian hypertension guidelines, transforming them into a single guideline and translating it into user-friendly hypertension algorithms for PHC workers. The adoption of this guidance represented a mindset shift in public health, from using guidelines developed by professional societies to using those co-created and owned by the city health authorities. This was one of the urban health initiative’s merits in São Paulo as it was unanimously acknowledged as an important driver for the success of this change. The updated guidelines and algorithms for service delivery in PHC were subsequently published [50, 51]. According to a representative of the São Paulo Cardiology Society, strong dissemination of the successful results of this standardized simplified approach has started and aims at promoting it at larger scale up to national level.

Two broader factors contributed to the success of these changes: firstly, the political will was strong in the city government leading to the introduction of a new guideline for NCD management in 2019 to impact their morbidity and mortality. As hypertension was one of its main focus areas, the simplification process of hypertension management offered a clear mandate to accelerate standardization at primary level. Secondly, weaknesses in primary health services for NCDs were identified that could be addressed. When asked why this guideline consolidation or primary care service optimization for NCDs did not take place prior to the initiative, interviewees mentioned a discrepancy between the theoretical framework and its practical execution. While many acknowledged the long-term need for guideline simplification, everyone regretted that no one truly took ownership to kick-start the process and introduce that. They also described the abundance of guidelines and solid legislation for the public health system in Brazil, while pointing at political, financial and/or operational level bottlenecks for improving service delivery. One of the main challenges seemed to be a general shortage of human resources in the health system and the limited motivation for operational duties among health system managers. The urban health initiative, with its strong focus on execution, truly addressed this bottleneck, as suggested by the following statements:

*“The point is that we don’t need another document. We don’t need another call to action. We don’t need to state what everyone knows. We just needed to have an effective intervention [referring to the urban population health initiative] and have metrics evaluated over time….” (Representative Cardiology Society of São Paulo).*

*“The initiative arrived at an opportune moment in primary care, launching the new protocols of NCDs, supporting their wide dissemination to all managers and health professionals in the municipality and focussing on continued support to the planning, execution and monitoring of actions.” (Representative, Municipal da Saúde, São Paulo).*

The political support for healthy schools occurred in a similarly change-ready environment, following the launch of the Ministry of Education’s programme “Saude na Escola” in 2007. While the City adhered to the policy, the intra-governmental collaboration between the Secretaries of Education and Health, was limited. The initiative aimed to catalyze such intersectoral collaboration, both within and outside the government, and created local ownership from the authorities as of its start. This enabled the initiative to support both Secretaries to align on joint agendas, by i) discussing the importance of intergovernmental collaboration; ii) developing a programme that integrated the ambitions and activities of both “Saude na Escola” and “Cuidando de Todos”; (iii) implementing the programme in selected facilities for children, students, youth and adults with infrastructure such as sport facilities, community centres and libraries, and by making them best-practice examples for other facilities. This programme included early screening, healthy lifestyle, physical activity and emotional care. The collaboration between government departments created a new connection between education and PHC facilities for young people, teenagers and their families [52].

## Discussion

The urban population health initiative primarily aimed at having a positive impact on the health of urban populations, and it did so by catalysing health system improvements, innovating health service delivery and engaging communities. Interventions include optimization of health system processes, continuous education of health workers, bringing health and care closer to people (e.g. in schools and workplaces) and advocacy. It also played a role in advancing health policy, especially in Mongolia and Senegal, by creating windows of opportunity through converging health burden and primary health problem statements (problem stream) with the politics stream within the contemporary policy framework and by fostering local policy entrepreneurs and creating evidence from the field that would offer arguments for change to policy-makers. The degree of influence the initiative exerted on policy reform varied between cities, yet a common set of enabling factors was identified.

### Enablers and challenges for the urban population health initiative to drive cardiovascular health policies in Brazil, Mongolia and Senegal

From a *problem* perspective, a different CV disease and risk profile was found in each of the cities. However, in each setting there seemed to be a sense of urgency to act upon the high CV disease prevalence. For Mongolia, a high CV mortality due to haemorrhagic stroke was observed and related to the high burden of hypertension and its underlying social and environmental determinants, including air-pollution, smoking and alcohol abuse. Especially in Ulaanbaatar, the awareness for hypertension and its associated risks had been increasing in recent years, and it became evident that a new approach was urgently needed to reverse the high burden of stroke in the city. The city major therefore welcomed the opportunities that the urban population initiative provided at the time. In Senegal, a relatively young age at onset of hypertension, a lack of awareness around CV disease, and insufficient, irregular access to medications were seen as key issues. The 2015 STEPS survey documented the size of the hypertension problem and further added to a momentum that had been building against NCDs, leading to the strong alignment of interest between the MOH NCD Division and the urban population initiative in 2018. In Brazil, increasing CV disease and obesity followed growing sedentarism and physical inactivity in urban populations. At the time the initiative approached the São Paulo city government, there were growing waiting lists for specialist examinations in the city, which the government intended to actively reduce by ramping up NCDs service capacity in the PHC system. The problems were mounting and susceptibility for the solutions provided by the initiative was therefore strong. The *policy* environment before the start of the initiative also differed, with Senegal lacking policies to improve lifestyle behaviour to prevent NCDs, or to address inactivity, tobacco use or salt consumption. In addition, the country was late in establishing an operational NCD Unit within the MOH. Mongolia, on the other hand, did not have tobacco control policies in place prior to the initiative, but it did have a comprehensive law on alcohol control. Brazil was stronger in its food regulations, based on the food labelling law of 2004. From a *politics* perspective, the national policy-making process was perceived to be relatively accessible in Mongolia with a centralized system and a single urban health authority covering a population of 3.3 million. Brazil on the other hand had a complex federal governance with more than 5500 municipalities, housing a population of about 213 million. Nevertheless, the political environment in all settings was described as quite susceptible to the goals and objectives of the urban population health initiative. When starting in São Paulo, the initiative was perceived as part of private sector’s corporate social responsibility and was considered more of an awareness raising project. This was probably due in part because Brazil had little experience with public–private partnerships in the health sector, and it resulted in longer lead times for the cross-sectoral alignment of goals and objectives. Whereas a similar scenario was observed in Mongolia, actors in Senegal were more used to foreign collaborations in health.

Common enabling factors and strategies were identified across settings that could overcome some of those external differences. The problem was well defined from the outset and in each location there was a sense of urgency to address CV disease, given its impact on both population health and the national economy. Focusing on hypertension, the initiative offered a well-defined entry-point and followed a stepwise path of i) situational assessment to clearly define and quantify the size of the problem and unmet needs; ii) co-design phase for defining needs-driven solutions and interventions; iii) implementation of interventions; and iv) progress monitoring and evidence generation on health outcomes and impact. While CV disease policies and health systems were different, similar needs were observed in each setting, such as the need to improve and simplify hypertension management and standardize algorithms of care, to enhance CV health education in schools and to optimize access to medicines. Based on local specificities, the initiative then responded to gaps in policy or service delivery, such as supporting the MOH in Dakar to consolidate its operational plan for NCDs, or by rather addressing inefficiencies in the execution of existing strategies in Brazil. Priority needs were always identified with local stakeholders and interventions to remediate those selected accordingly. This co-design approach enabled the initiative to deliver successful outcomes in each of the cities, both in policy reform and in population health. On the political level, political alignment was identified as the key determinant for success (Table 5). Subsequent factors and strategies enabling success spanned across the problem, policy and politics streams and meaningfully connected them. Following an approach based on alignment, co-creation and support to local partners and strong local ownership in each country, this global initiative was able to achieve both improved health outcomes and influence health policy. As such it succeeded in creating an environment for sustainable change.

**Table 5:** Factors that enabled the urban population health initiative to work towards the introduction of policies with a proven impact on health

• Clear national governance with goals and strategies that are aligned to those of the initiative	
• Strong local and global partner(s) who support the implementation by the local partners	
• Global expertise contributing to the partnership where needed, e.g., to deliver evidence-based strategies	
• Ensuring local ownership as from the start, by taking a consistent approach to co-create and co-design solutions and interventions with all relevant stakeholders	
• Selectively fund innovative or more ‘risky’ interventions for which local resources are unavailable	

### Multiple streams analysis – international experiences

The current multiple streams analysis adds to the limited scientific literature on CV disease policy assessments in LMICs. The existing literature applying the Kingdon framework in LMICs, is mainly limited to Iran, Turkey, Russia, Nepal and Kenya, and features policies around hepatitis C, HIV/AIDS, tobacco and the ‘Health in All Policies’ strategy, to assist decision-making for policy makers [19, 30, 53–56]. We found no comparable study for Brazil, Mongolia or Senegal, but even though the disease focus in our study is different than those in existing literature, some factors influencing agenda-setting were found to be similar (e.g. lack of awareness and information, lack of diagnostic facilities and high costs generated by the disease). On the policy level, driving factors that were similar between the literature and our study were campaigns to increase awareness in schools, universities and parks and influence by multilateral agencies (e.g. 2030 Agenda for Sustainable Development). International influence also seemed to have played a role in the political prioritization of tobacco control in Turkey [19]. Another piece of work used the Kingdon framework to explain Russia’s struggle to manage HIV/AIDS, concluding that the limited political commitment and an increasing trend towards state autonomy marginalized the civil society’s role and interests in policy making. The countries assessed in our study showed, however, a more liberal way of collaborating between government and civil society, thereby giving the latter greater opportunities to contribute to the policy discussion. Especially in Senegal, patient and professional organizations were considered important feedback and input mechanisms for government decision-makers, and they played a major role in driving NCD policies, including those related to diabetes and renal disease (Annex 1).

### Limitations

The main limitation of the presented study is that the Kingdon framework was not applied to achieve an exhaustive in-depth analysis but it provided more general guidance for data collection and analysis. Following the relatively broad scope of work here presented and the focal study question of the role of the urban population health initiative at the policy level, the depth of our sub-studies is more limited than it might have been if we would have focused on one policy development in a single country. We nevertheless aimed at presenting a complete view of the most relevant policies related to CV health in each of the three cities where this initiative was rolled out. Related to the broad study design and the main study question, the reviews of the peer-reviewed and grey literature did not take place in an exhaustive manner. Information was gathered primarily from initiative documents, validated in interviews and, where gaps arose or contradictions were observed, the wider peer-reviewed and grey literature would be consulted. Any separate in-depth study on a single policy adaptation in one setting, would be expected to have added depth.

For external validity, although our study shares similarities with outcomes reported in other studies, the case studies are specific to the three geographical areas – Brazil, Mongolia, and Senegal, and therefore the findings have to be applied to other contexts with caution. Regarding internal validity, a potential bias in our study could come from the sample of interviewees and participants, as it was impossible to engage all representatives in the field of policy-making, but we rather focused on feedback from stakeholders that were close to the initiative. Moreover, we cannot exclude a history bias; the documents of each case study ranged a wide span of time, and events that may have impacted the country’s politics and the policy level that we have reported on may have been lost or over−/under-emphasized.

## Conclusions

Using an approach of alignment, co-creation, local ownership and support to local partners, our urban population health initiative was able to achieve its primary goal of increasing BP control in urban populations and influencing health policy to augment the potential for creating sustainable change. The approach enabled countries to tailor the initiative to their needs, both at policy and operational level, and to achieve remarkable changes in population health. These implications and key lessons are widely applicable to other complex multi-disease, multi-sector and multidisciplinary implementation projects; they provide other initiatives guidance on implementation and stakeholder management. Our study demonstrates the interconnected nature of service delivery and policy making in health, as well as the influence that concrete interventions can have on policy dynamics. Our experience in three countries demonstrates that, although the history of policy and politics does play a role in shaping policy-oriented initiatives, it is often the most relevant and concrete needs that drive sustainable change and offer a successful entry point into the political environment.

### Supplementary Information

**Additional file 1.**

## Data Availability

All data relevant to the study are included in the article or uploaded as supplementary information, except for the raw interview data. The interview data generated and analysed during the current study are not publicly available because it is practically impossible to completely anonymize all audio materials and transcripts. However, anonymized data are available from the corresponding author on reasonable request.
