# Supplementary material for: Assessing the contributions of an urban population health initiative to shift political priority towards cardiovascular health: three case studies from Brazil, Mongolia and Senegal
Source: BMC Health Serv Res. 2024 Jan 4;24:16. doi: 10.1186/s12913-023-10432-8 (PMC10768224; doi:10.1186/s12913-023-10432-8)
Supplement: Supplementary file 1 — Additional file 1. [file 12913_2023_10432_MOESM1_ESM.docx]

**Annex 1: Kingdon streams Mongolia**

**NCD problem stream Mongolia**

***Geopolitics & demographics***

In 1992, Mongolia adopted a new constitution transforming from a closed single-party Communist state to a multiparty democracy. This transition was accompanied by relative political stability, a step-wise integration of free-market reforms and rapid urbanization leading to half the Mongolian population living in the Capital, Ulaanbaatar. A new openness towards non-healthy foods together with a dramatic change in lifestyle, i.e., from pastoralist to sedentary urban has, however, contributed to a growing NCD burden. On top of that, excess salt consumption, smoking and drinking are widespread with more than half of the male population smoking and a high degree of binge drinking among the alcohol consumers being observed. Extreme air pollution further fuels the NCD burden; approximately 30% of the population in Ulaanbaatar is living below the minimum wage and is therefore resorting to burning coal in winter which renders Ulaanbaatar one of the most polluted capitals in the world.

***Surveys and NCD epidemiology***

The extent of the national NCD burden was revealed for the first time in full detail by the first National NCD STEPS survey published in 2006 and conducted by the Ministry of Health and its Public Health Institute together with WHO.^1^ Although previously, several small-scale surveys were conducted in Mongolia in relation to NCDs (including national surveillance efforts in nutrition, salt intake and global school-based student health), the results were not representative of the national situation regarding the NCD burden and associated risk factors.^2-4^ The Mongolian NCD STEPs survey was therefore designed to provide information on the prevalence of the major NCDs and their risk factors, in relation to socio-economic and behavioural factors, across the whole Mongolian population to establish nationwide comprehensive baseline information. The 2006 STEPS survey found that the prevalence of hypertension among Mongolians aged 15–64 years was 28.1%, while the prevalence of diabetes was 8.2%, an increase of 5% since 1999.^3^ The survey also found that 91% of the surveyed population had at least one risk factor for developing NCDs and that 21% had three and more risk factors ^1^. Two subsequent STEPS surveys were conducted in 2009 and in 2013 demonstrating that the prevalence of hypertension in Mongolia persisted near 28% between 2006 and 2013 but that the rate of hypertension control sequentially declined from 21% in 2006 to 12% in 2009 and to 7% in 2013 (however, 2013 numbers are disputed – see policy section main article).^5 6^ The 2013 STEPs report also revealed that, although some improvement in BP screening since 2009 was observed, an estimated 27% of individuals never had their BP measured. Moreover, among individuals previously diagnosed with hypertension, 72% were not on medications and 21% were on medication but with uncontrolled BP levels.

In 2011, a first national baseline assessment on salt consumption was conducted, revealing that the average daily salt intake, based on 24 hour urine samples from a representative national sample (n=1,027), was 11.06 ± 5.99 g, more than double the WHO five grams recommendation.^7^

***Health system structure***

Mongolia’s health care system still operates by the Semashko system^8^ – an artefact from its association with the Soviet Union. Heavily based on hospital care, the system is ill-equipped for preventive medicines approaches and NCD management. Preventive public health measures seem to be given very little priority. Consequently, Mongolia’s public spending for NCDs is dominated by inpatient care and hospital-based specialist outpatient services suggesting an inefficiency in resource use ^9^. Hypertension and CV disease investments seem to have predominantly focused on the tertiary health system, with little funding and training for PHC. Several respondents emphasised the high turnover of doctors at PHC that is leading to poor institutional memory at primary care centres. PHC seems to be more of a temporary training opportunity from which staff moves on to secondary or tertiary care. Low levels of accessibility and affordability of generics have been confirmed by reports from the Ministry of Health. Moreover, the cost of a 30-day supply of these drugs can range from 0.4–2.7 days wages depending on whether purchased in public or private sectors.

***Operational challenges in health care***

On operational level, the predominant issue was described to be the lack and/or quality of data collection and analysis. Different actors have, over the last several years, recognized that there is an urgent need to create an integrated health information system that would enable patient-centred continuous quality improvement of healthcare delivery and outcome. For a long time, only piecemeal and incremental solutions were available, focusing on specific disease areas and certain health issues creating silos of software solutions at healthcare centres and facilities around the country. The first-ever coordinated attempt to establish an integrated electronic medical records at all levels of Mongolian healthcare system was to be an integrated electronic medical records called eHealth, financed by approximately 20% of a US$20 million soft-loan from China and implemented by Huawei Technologies Co. Ltd. of China. However, because of shortfalls in creating patient-centric workflow, outdated technology usage, implementation errors and controversy from the very beginning (i.e. bribery at the Ministry of Health level during the tender procedure) eHealth failed to become the preferred solution within the Mongolian healthcare system in the long-run. At the same time, another project, also called eHealth, financed by the US$20 million soft-loan from the World Bank, initiated its implementation to develop a vertical infrastructure to assemble nationwide health data into an integrated database – the integrated vertical HIT system project. On this front, the Onom Foundation works closely with the World Bank and the Government of Mongolia. The latest attempt of enhanced health data integration was made by the urban population health initiative aiming to integrate four hypertension patient registries (eHealth, ePrescription, mHealth and Excel-based hypertension registry) into a single mHealth registry or at least have a common interface for automatic data exchange. However, there has been no capitalization of those efforts owing to technical issues.

**NCD policy stream Mongolia**

***General NCD programs, strategies and policies***

Since 2000, the government of Mongolia has demonstrated a timely and coordinated response to NCDs.^10^ In 2006, the National Program on Integrated Prevention and Control of NCDs was introduced,^11^ followed by the GER Nutrition Guidelines in 2007. In 2010, a National Strategy on Healthy Diet and Physical Activity (2010–2021) was launched as well as a Health Education Strategy and a National strategy on Information Education and Communication to Promote Healthy Behaviours. In 2011, clinical guidelines for arterial hypertension were established, while 2012 saw the adoption of a food law and a National Program on Healthy City, District, Workplace and Schools (2012 – date). Preventive measures in the more recent years (i.e. 2017–2020) include the Developed ‘Nutrition Education 1–6 grade’ student, teacher books and tools, introduced on national level together with a law for mandatory annual check-ups (Table 1). On healthcare level, both the MoH and the National Health Insurance Fund (NHIF) increased their annual direct budgets for PHC. The NHIF also increased the number of subsidized antihypertensive medications which led to a 2% increase in average cost coverage. Moreover, new hypertension guidelines and a primary care algorithm were approved and implemented in all healthcare clinics in Ulaanbaatar.

***Millennium Challenge Corporation project***

One of the earliest initiatives to tackle hypertension from a multidisciplinary approach was the USA Millennium Challenge Corporation (MCC) program conducted together with the Mongolian Government and based on a five-year contract signed in October 2007. Of the total funds, $42 million was devoted to a health component focused on NCD capacity strengthening, behaviour change and strengthening health systems. Specifically, the health component aimed at increasing the adoption of behaviours that reduce NCDs among target populations and improve medical treatment and control of NCDs. Implementing partners were the MoH, EPOS Health Management, Onom Foundation – the local implementation partner for the urban population health initiative – and other local NGOs. Informants stated that the MCC project was the one that really brought the NCD issue into wider national attention. According to the informants, training was a major activity within the MCC project, resulting in 50% of all of Mongolian health care workers being trained in non-clinical disease care. The 2009 and 2015 STEPS surveys were funded and conducted by the MCC project and the first hypertension guideline was developed. Benefits of the project regarding CV disease were observed on two main levels. First, hypertension care improved; according to the STEPS reports, stroke mortality decreased from 2% to 1.5%. Surprisingly, upon initial analysis, this change in mortality did not manifest in hypertension control changes as suggested by the STEP surveys (12% in 2009 vs 7% in 2013). However, re-analysing the data, it was discovered that a mistake had been made and that hypertension control had gone up after all. Second, people’s awareness of risk factors, especially regarding healthy diet and physical activity, were perceived to have increased owing to the extensive campaigning activities of the MCC project.

***Tobacco and alcohol***

In 2000, an alcohol control law was adopted and updated in 2009, and a National Program on Prevention and Control of Alcoholism was created in 2003. Mongolia had also developed strong tobacco policies to reduce demand. In fact, informants stated that tobacco-consumption had seen some of the most prominent and potent NDC policy-level changes. In 2005, following a global youth tobacco survey the previous year, a tobacco control law was adopted and updated in 2012. In this update, the existing law was revised to support larger health warnings, banning cigarette sales in vending machines and over the internet as well as banning smoking in all public places and increase excise tax on tobacco by 2%.^12^ According to an informant, a pharmacist and owner of one of the biggest national pharmaceutical companies *cum* parliament member was an important advocate for this update in 2012, recognizing the excess second-hand exposure outside.

***Salt***

Following on from a WHO regional consultation on salt reduction in Singapore in June 2010, the MoH in collaboration with MCC project, WHO and the National Center for Public Health in Mongolia, organized a salt reduction consultation meeting in Ulaanbaatar. An inter-sectoral working group was established and a series of meetings were conducted with government officials and technologists from bread companies and the meat industry. The objectives of these meetings were to raise awareness of the burden of NCDs associated with high salt intake, introduce the WHO recommendations on salt reduction, and invite the food industry and other stakeholders to collaborate on efforts to reduce salt intake across the population. An action plan to establish a national baseline on salt consumption patterns and implement a series of pilot initiatives to reduce salt intake was developed and endorsed by all of the key stakeholders. A pilot Pinch Salt intervention to reduce salt consumption of factory workers was undertaken in Ulaanbaatar city between 2012 and 2013, and was associated with a reduction of 2.8 g of salt intake. Ongoing food industry initiatives have led to significant reductions in salt levels in bread, and companies producing processed meat have indicated a willingness to reduce salt. Relevant stakeholders have also supported the campaign by participating in annual World Salt Awareness Week events since 2012. The Mongolian government has identified the tackling of NCDs as a high priority health objective for its population and has already successfully implemented its first National Programme for NCD risk factors during 2006–2013.^10 11^ The national salt reduction strategy for Mongolia – implemented from 2015 to 2025 – is clearly focused and unites all players: government, the food industry and community, in one ultimate goal – to reduce salt intake in Mongolia by 30% by 2025. The salt reduction initiatives focused on employers and the food industry and both have been viewed successful in lowering intake. A return of investment analysis in 2017, by the UN Inter-Agency Taskforce, further supported the case for salt policies, demonstrating that compared with other interventions pursued for tobacco control, alcohol and CV disease clinical interventions, salt interventions have the highest return on investment.^13^

**NCD politics stream Mongolia**

***Bi- and multilateral funders***

There have been major externally funded, government-led projects to support the general health care system and strengthen the NCD national response. The World Bank Group & United Nations Development Programme (UNDP) were among the first funders in the new post-communist era. Since 1997, they have been collaborating with the Ministry of Finance on a 10-year poverty alleviation national plan. Since 1997, they have invested and mobilized more than $470 million to date including the $20M for ehealth software development efforts with the MoH. The UNDP has also done various relevant health projects in Ulaanbaatar including a prevention/awareness campaigns and SMS mobile apps, funded micro-insurance product development with various insurance companies.

Another international donor was the Luxembourg Development Cooperation (LuxDev) funding a 11-million EUR project that was running from 2011–2016, aimed at improving health services in the areas of CV disease and mother and child health care. During the same time period (2012–2016), the Asian Development Bank (ADB), launched a health sector development project. Being Mongolia’s largest external financier to the health sector, ADB provided loans totalling $29.9 million for hospital infrastructure, eight technical assistance operations and three grant projects. It typically funds infrastructure, health policy development and other core government/clinical focus areas.

The USA Millennium Challenge Corporation program and the Government of Mongolia signed a five-year, $284.9 million contract in October 2007. Of the total funds, $42 million was devoted to a health component focussing on NCDs including a large scale initiative on hypertension. This initiative represents one of the earliest initiatives to tackle hypertension from a multidisciplinary approach.

More recently, i.e., in 2019, a ‘Resolve to Save Lives’ grant has been awarded to Onom Foundation, for the national rollout of the activities initiated by the Better Heart Better Cities initiative.

***Involvement of national media channels***

Among the bi- and multilateral funders, the MCC stood out, in the conversation with informants, with regards to the intensity of campaigning and inclusion of the local and national media bodies and lobbying groups including the Mongolian National Broadcaster for television and radio. The Mongolian National Broadcaster was responsible for all major campaigning of the MCC program, including for the prevention of alcohol and tobacco consumption. According to an informant, they have organized frequent events, discussions with, e.g., parliament members and experts and offered their television and radio platforms for broadcasting. This seems to have contributed significantly to the sustainable education of the wider Mongolian population on CV disease risk factors, especially pertaining to healthy diet and physical exercise.

**Kingdon streams Senegal**

**NCD problem stream Senegal**

***Epidemiology***

Senegal is undergoing an epidemiological transition. Historically, the countries’ public health priorities were infectious diseases – above all malaria, tuberculosis and HIV/AIDS. Having made large progress on this front, Senegal is losing ground against an ever increasing burden of NCDs, a main contributor being the globalization of lifestyle and dietary habits and a relatively young population, that is, however, largely unaware of NCDs and their risk factors. In addition, the international funding community, i.e., bilateral and multilateral entities, has not caught up to the NCD-related needs in Africa and is often still focused on ‘the big three’ infectious diseases, maternal and child health while actions against NCDs seem to be poorly funded in Senegal and elsewhere on the continent.

***Surveys***

Most informants pointed towards a strong anecdotal level of evidence around NCDs that first raised attention to this class of diseases, prior to any comprehensive survey. An increasing frequency of more severe complications, especially heart attacks and strokes was, for instance, noted within families and at the health care level. In health facilities, the main reasons for consultation or hospitalization are NCDs, especially diabetes, hypertension, pulmonary disease, cancer or kidney diseases. NCDs were also highlighted by smaller studies or in certain high-prevalence regions of Senegal. However, the extent of NCDs and their risk factors was not sufficiently understood until the 2015 national STEPS survey^14^ was conducted delivering a data set that explained the problem in detail and demonstrated the significance of NCDs. Hypertension was found highly prevalent, with almost one out of three persons (29.8%) between 18–69 years, suffering from the condition and with less than half of them (46%) being aware of having it.^14^ In addition, only 17% of the hypertension patients reported taking antihypertensive therapy and 8% achieved BP under control (unpublished data). Also, the prevalence of arterial hypertension increases with age (from 15.3% of those aged 18 to 29, to 64.2% of those aged 60 to 69). Stroke, is considered today as the fifth major risk of death in Senegal. Its incidence is expected to continue to increase and double by 2030. The most recent NCD report from the WHO estimated that, in 2016, NCDs accounted for 42% of all deaths in Senegal – 17% of all deaths were attributed to CV diseases alone.^15^

***Health care system and access***

Almost two-thirds of all Senegalese are lacking access to health insurance and are facing relatively high healthcare costs. Despite Senegal launching a Universal Health Insurance Program in 2013, coverage rates remain far from the government’s target in 2017 that was 75%. It has been documented that the healthcare costs currently remain high for the Senegalese population and while UHC has been instituted coverage remains low.^16^ Direct spending on the prevention of NCDs has not traditionally factored as a prominent item in public health expenditures.^17^ Senegal spends 4.7% of total GDP expenditure on health^18^ and poverty rates remain high in Senegal, affecting 46.7% of the population.^16^ Patients often face barriers to accessing and following treatment, for example affordability and the patient’s capacity to pay for the care they need.

In the majority of health districts, essential drugs for high blood pressure were available at the primary level through the distribution circuit of the national supply pharmacy (Pharmacie Nationale d'Approvisionnement; PNA). However, these anti-hypertensive drugs suffered from frequent shortages in health centres and health posts. According to the National Agency of Statistics and Demography (Agence Nationale de la Statistique et de la Démographie; ANSD) converting enzyme inhibitors (enalapril) were only available in 3% of cases, 1% for thiazide diuretics, 3% for beta blockers (atenolol) and 33% calcium channel blockers (amlodipine).^19^ Despite most antihypertensive medicines appearing on the essential medicines list, stock outs and their costs were major barriers in Senegal.^20^

***Guidelines***

The Ministry of Health and Social Action (Ministère de la Santé et de l’Action Sociale; MSAS) in collaboration with the Chair of Cardiology, set up in 2016, a collection of protocols for the standardized management of hypertension for the entire health system. Before, the MSAS had a few protocols on diabetes and other NCDs – however, they were elaborated by second- and third- care practitioners and showed to be not easily implementable owing to their complexity and resource intensity. However, healthcare providers in health districts in the Dakar region have not benefited from the dissemination of these protocols. The only support tools available in the points of service of the health districts of the department of Dakar were brochures distributed by the drug laboratories which promoted their products. Despite the availability of these protocols, the primary level (i.e. health centres, health posts) was left to itself as part of the management of hypertension. Patients were generally taken care of by staff who did not have all the technical tools to ensure quality care.

***NCD programs***

According to several informants, some health districts including Dakar West, benefited in 2014 from Sanofi Laboratories, Diabetes Hypertension Centers (CDH) that aimed at strengthening the supply of quality care for hypertension. The objective was to set up a decentralized reference centre for the management of diabetes and hypertension, which was to polarize all the health centres and health posts in the health district. However, these CDHs have never been able to implement their roles of supervision vis-à-vis other polarized structures and polarization of the counter-reference system. The majority of healthcare providers had not received continuous training on the new guidelines for the management of hypertension. The rare training courses available were organized by the laboratories in the form of postgraduate courses. Some cardiology specialists have benefited from training often through conferences, training abroad or during forums. The Sanofi programme on diabetes and hypertension took place on tertiary healthcare level and was perceived to have been left without continuum and wider integration into the system.

The ‘mDiabetes’ prevention work of WHO, on the other hand, was perceived an important contribution. However, taking place as annual short intervention during Ramadan it was not seen as a comprehensive type of intervention in its own right.

**NCD policy stream Senegal**

***National strategies & programs***

In Senegal, the National Health and Development Plan 2009–2018 (Plan National de Développement Sanitaire; PNDS) ^21^ already remarked on the expensive care of NCDs and the heavy burden for Senegalese households. The plan was indeed calling for increased attention from the Ministry of Health and Social Action (Ministère de la Santé et de l’Action Social; MSAS) and an inventory of current local capacities with regard to human resources and equipment to identify gaps and develop an action plan for the operationalization of treatment units and to make treatment more accessible in each region.^21^ Yet, prior to 2014, there were barely any policies and strategies in place that would help the increasing NCD burden.^22^ Nutrition, for instance, has had high political attention in Senegal since 2001, but mostly from an undernutrition perspective.^23^ This, however, changed dramatically around the same year, when the NCD Division was created within the MSAS (see Supplementary information ‘Politics stream Senegal’ for detailed information), developing an integrated National Operational Plan for Cardiovascular and Metabolic Diseases 2017–2020.^24^ The document served to fight against priority NCDs; at the same time, this plan also targets risk factors including smoking, sedentary lifestyle, unhealthy diet, alcohol and air pollution. The subsequent National Health and Social Development Plan (Plan National de Développement Sanitaire et Social; PNDSS) 2018–2028^25^ has a corresponding strong orientation towards NCDs with an action point 46, dedicated to the ‘preventive and curative care of NCDs’ based on following strategies: (i) put in place a multisectoral framework for the fight against NCDs, to develop preventive actions such as physical exercise, the fight against smoking, alcohol abuse and unhealthy diet; (ii) develop the Public Private Partnership (PPP) to mobilize resources; (iii) promote the mobilization of endogenous resources; (iv) strengthen awareness among populations; (v) strengthen the health system at all levels of the pyramid, with a view to early diagnosis and appropriate and efficient management of NCDs and; (vi) develop screening, surveillance, operational research, monitoring and evaluation of interventions on NCDs.^25^

***Tobacco & alcohol***

In 2014, Senegal saw also changes with regard to tobacco policies. As assessed by the WHO MPOWER scheme – a tool to help countries implement WHO FCTC demanded reduction measures – Senegal has introduced three best-practice policy interventions since 2014^26^ i) offering help to quit tobacco use; ii) warn about the dangers of tobacco; and iii) enforce bans on tobacco advertising, promotion and sponsorship.^27^ Senegal attained best practice in each of those measures by 2016. By then, the country had introduced a comprehensive ban on all tobacco advertising, promotion and sponsorship, including at the point-of-sale. With regards to their cessation services for tobacco users, Senegal was, as reported in 2019, the only low-income country that offered full cessation support.^27^ In 2014, when Senegal adopted its Tobacco Control Act, especially tobacco cessation was seen as a national priority and had strong backing from the Health Commission of the country’s National Assembly and the Chair of the Health Commission, Awa Dia Thiam. The MSAS has since established a national toll-free line where trained counsellors give advice on smoking cessation. In addition, a National Tobacco Control Strategic Plan 2018–2022 was developed by the National Tobacco Control Program that described all available cessation services. The law to restrict smoking in public areas was, according to informants, strongly advocated by a medical doctor specialized in cancer – who is heading an anti-tobacco lobby group of smaller medical associations and has ties to the government.

With regard to alcohol consumption, however, there seems to be no written national policy or action plan according to WHO.^28^ However, there is an excise tax on beer/wine/spirits and legally binding regulations on i) alcohol advertising and product placement; ii) alcohol sponsorship and sales promotion; and iii) alcohol advertisements and containers are in place. Moreover, there is national government support for community action and some restrictions apply for on-/off-premise sales of alcoholic beverages.^28^

***Cancer***

Since October 2019, Senegal's government offers free chemotherapy in public hospitals to women suffering from breast or cervical cancer. The government further committed to reimburse 60% of the costs for other types of cancers.^29^ According to informants, this success has been largely driven by the civil society, namely health providers and academics that have observed the rise in particularly cervical and breast cancer and the Senegalese anti-cancer league (Ligue sénégalaise contre le cancer; LISCA). The same year Senegal also established a National Cancer Control Programmes, cancer management guidelines, made available three different types of treatment services (i.e. surgery, radio- and chemotherapy) and started a breast cancer screening program and an early detection program for cervical cancer.^30^

***Diabetes and renal disease***

An operational strategy for diabetes was covered by the National Operational Plan for Cardiovascular and Metabolic Diseases 2017–2019. In addition, blood glucose was measured as part of the 2015 STEPS survey. Insulin, metformin and sulphonylurea are the available medicines in primary care facilities, and renal replacement therapy by dialysis is being offered as a procedure.^31^ According to informants, insulin and diabetes care has become free of charge in 2004 owing to pressure from advocacy groups including the Association of Diabetes Patients (Association Sénégalaise de Soutien aux Diabétiques/ASSAD) the Mark Sankalé Diabetes Center (national referral centre for diabetics).^24^ An equally big policy achievement was stated to be the subvention for dialysis where increased and free access was ensured after a period of high mortality from renal dysfunction and low and expensive access. Equally, this policy change was driven by an associations of people affected by kidney disease. Now, 80% of those affected were stated to have access because the government changed the policy and they increase the number of dialysis machine in the country.

***Hypertension***

Several of the most recent updates on national NCDs policy- and systems level pertained to hypertension. In 2018, the NCD National guidelines were translated into one single algorithm of care for clinical decision support in the management of hypertension in primary care. A continuum of care was established with national guidelines and primary care algorithm for hypertension and a defined patient pathway. Nurses of primary health posts are now able to refer hypertension patients to 2nd or 3rd care (task shifting policy) and any adult ≥18 is being screened. Moreover, the change in algorithm was accompanied by an update in the national policy enabling community health workers to screen for BP, refer patients for confirmed diagnosis, and for following up patients once diagnosed with BP. As second major advance was observed in access to hypertension medicines. While amlodipin and captopril were previously to be prescribed in primary health centres a revision of the national drugs list in 2018 changed this situation and allows for those medicines now also be prescribed at lower level facilities such as in community health posts. Finally, the World Hypertension Day was celebrated for the first time in 2018 and then again in 2019.

**NCD politics stream Senegal**

***Internal political drivers***

NCDs have come onto the political stage during the presidency of Abdoulaye Wade (2000–2012) under whom special attention was paid to NCDs in the National Health and Development Plan 1998–2007 during its last three years (Plan National de Développement Sanitaire; PNDS). The subsequent PNDS 2009–2018 explicitly referred to the growing double burden of diseases and noted the magnitude, disabilities caused and heavy expenses caused by treatment of NCDs, therefore calling for increased attention from the MSAS.^21^ Moreover, this document was already suggesting a survey on the prevalence of risk factors for NCDs with the WHO ("STEPS" survey) in order to orient strategies in the direction of NCD prevention. This focus on NCDs has only accelerated under the current President Macky Sall who has been in office since 2012. The national socio-economic policy relates to the vision of President in his ‘Emerging Senegal’ plan (Plan Sénégal Emergent; PSE), a ten-year strategy for the period 2014–2023 for an emerging Senegal by 2035. This should be achieved through three strategic axes including one on human capital, social protection and sustainable development. Health is considered as an essential input to move towards emergence and development. While the first level of reference is the PSE, the second level of reference is the National Health and Social Development Plan (Plan National de Développement Sanitaire et Social; PNDSS) 2019–2028.^25^ The particularity of Senegal is that NCDs are a featured priority at the level of the Ministry with orientation for the fight against NCDs given by the PSE at the strategic level of health development and at a more specific level in the National Operational Plan for Cardiovascular and Metabolic diseases. So, suddenly there was a strategy to be implemented to mobilize resources at the domestic and international level to fight against NCDs.

***NCD Division in the MSAS***

In April 2013, an NCD Division was created within the MSAS with a unit focusing on CV diseases. Different reasons for the creation of the NCD Division were cited by the informants; however, the reasons named are predominantly based on internal drivers in spite of a meagre international NCD funding landscape. The formation of the division seemed to have been driven by a mix of factors including civil society pressure and increasing awareness of the consequences of the populations’ lifestyle change and the fact that NCDs (and their economic costs) were increasing dramatically. Moreover, the Minister of Health at the time –Awa Marie Coll-Seck – was a professor of medicine with a high awareness of the ongoing academic and medical discussions around NCDs and was named by multiple informants as key advocate. She also played an instrumental role in orchestrating a National Operational Plan for Cardiovascular and Metabolic diseases. One of the first tasks of the NCD Division was to drive a STEPS survey (in alignment with the suggestions outlined in PNDS 2008–2018) with the help of WHO and the involvement of specialists (cardiologists) and organizations working on NCDs, e.g., the Senegalese cardiology society, the Association of Public Health Workers and medical doctors. The STEPS survey was to offer a detailed description of the national NCD situation and to help building a national strategic plan. Despite a change across ministries around the second half 2017 – including in the MSAS team, the momentum was maintained and the creation of the NCD Division, seems to have attracted the interest of different NGO’s, associations and other organizations working in the NTD arena, according to informants.

***Role of non-state actors***

Several informants emphasised the importance, dynamicity and communication savviness of patient groups, medical doctors and civil society organizations in the fight against NCDs in Senegal. Many changes seem to have come from patients directly affected by NCDs that formed into groups. Civil society groups were perceived to have a strong position when they are large associations and organizations. Diabetics, for instance, were the first big group to get organized into a diabetics association (Association des Diabetiques; ASSAD). Two to three decades ago, ASSAD made a lot of advocacy in order to get insulin and diabetes care cheaper or free. Indeed, the president of ASSAD is (a journalist) is the same time the president of the national group of journalists that are covering public health, and especially NCD-related areas, therefore exerting a significant influence in the NCD arena. The informants stated that also the introduction of free medical treatment for cervical and breast cancer was mainly a civil society achievement, driven by health providers, professors and Senegalese anti-cancer league (Ligue Sénégalaise Contre le Cancer; LISCA), raising the problem to the media and government. The same pattern was seen for kidney failure, a major cause of mortality a few decades ago. While at the beginning, only very few dialysis cabinets were available in the country and treatment was barely affordable, access today has dramatically improved thanks to the advocacy of an association of renal failure patients (Association Sénégalaise des Hémodialysés et Insuffisants Rénaux). The civil society in Senegal is working directly with a group of deputies for health in the National Assembly to improve law around health. There is therefore a direct line of communication from the civil society to the government, with the deputies in the national assembly having voting powers and representing the civil society.

***International cooperation***

There did not seem to be a large multilateral influence to the NCD policy and politics in Senegal, except for the first United Nations high-level meeting on NCD prevention and control that took place in 2011 and helped to increase awareness and political will across the globe including in Senegal. However, the Belgian Development Cooperation emerged as a prominent early bilateral donor. According to informants, the Cooperation has been supporting the MSAS for close to five decades in developing the PHC system and are today still supporting the government in strengthening the overall health care system and infrastructure. More recently, they were spearheading the creation of harmonized guidelines for six priority NCDs, including CV diseases and diabetes, working with the MSAS and the NCD Division and PATH finishing in 2016.

**Kingdon streams Brazil**

**NCD problem stream Brazil**

***Burden of disease***

According to informants, NCDs increased – somewhat silently – in Brazil over the last 30 years accumulating, at some point, into a very costly problem in terms of quality of life as well on healthcare financial level. Today, NCDs account for 74% of all deaths in Brazil, being the leading cause of mortality in the country, with CV diseases accounting for 28% of those deaths.^15^ According to population-based surveys representative of cities and states, the prevalence of hypertension may be anywhere between 23% and 30% among the adult population in Brazil depending on region.^32^ Data suggested that more than 80% of those may be aware of their condition.^32^ However, a comprehensive national estimate of prevalence still does not seem to be available. Access to hypertension medicines has been reported to be high; yet, studies suggest that between 35% and 55% of the treated patients do not have BP control.^32 33^

***Risk factors***

Changes in eating habits and the sedentary lifestyle of the Brazilian population caused an increase in overweight individuals and obesity and a decrease in the incidence of malnutrition. A mere 15% of the population pursue regular physical activity and only 18.2% of the adult population follow the ‘five servings of fruit and vegetables on five or more days per week’-recommendation. Moreover, approximately one third of the population consume high fat foods (34%) and soft drinks (28%) on at least five days a week.^34^ After the USA, Brazil is has the highest consumption of sugar per capita (600 kcal/day).^34^ Consequently, the prevalence of obesity in Brazil almost doubled within a 10-year period, increasing from 11.8% in 2006 to 18.9% in 2016. Even more dramatic was the increase in the overweight population from 18.5% to 50.1% and from 28.7% to 48% between 1974 and 2009, for men and women, respectively, according to the biannual national Vigitel survey on NCD risk factors.^35^ An additional risk factor is the high national salt consumption with an average consumption of 12 g of salt per day for adults – more than double the WHO recommendation. The main source of this daily salt intake are salt and salt-based condiments that are added to meals representing 76% of daily salt intake, according to the Brazilian Household Budget Survey 2008–2009.^36^

***Health system***

The Brazilian Unified Healthcare System (Sistema Único de Saúde; SUS), established 1989, is one of the best-known Universal Health Coverage models in Latin America striving to provide access to healthcare to the entire population. The SUS comprised of both public and private health care institutions and providers is responsible for most primary care services that are financed through contributions from the federal, state and municipal budgets. Brazil put in place a strong policy framework to tackle NCDs, including hypertension, and significant efforts were made to strengthen the capacities and coverage of primary healthcare under the SUS. Nevertheless substantial challenges remain, in terms of unmet population health needs, lack of patient-centeredness of care, inefficient care pathways and referrals quality.^37^ Based on available official data, it is estimated that more than 75% of the country’s population depends exclusively on SUS for health care;^38^ however the use of the services does not reach all individuals for all health conditions. All publicly financed health services and most common medications are universally accessible and free of charge, however, for those with private health expenditures, out of pocket health expenditures remain high at approximately 57.8%.^39^ Moreover, although, since the 1990s, health outcomes have generally improved for Brazilians, there is concern that NCDs and overall health will be impacted in the wake of the country’s recessions. In 2016, the government passed an amendment that froze the federal budget, including health spending to its 2016 levels for the next 20 years.^40^ Already, key social protection programs such as Brasil Sem Miseria (Brazil Without Extreme Poverty) and the Bolsa Familia (Family Allowance Program) have had cuts resulting in over one million families being excluded.^41^

Several studies have looked at the care pathways of hypertensive patients in the city of São Paulo. Most are carried out in the public healthcare sector and focus on patients already diagnosed with hypertension. Even among these patients, who are supposed to have relatively good access to care, the levels of treatment adherence are very low (only around 20% take medication on a regular basis).^42^ Consequently, only 35% to 50% of patients under anti-hypertensive treatment have their blood pressure controlled.^42 43^ Studies indicate that even after hypertension-related hospitalization, adherence does not improve much.^44^ These insights suggest that the expansion of population coverage and primary healthcare delivery infrastructure that happened in recent years under the reforms of SUS has not yet translated into improved health outcomes for people with chronic health conditions. There are several bottlenecks to be addressed regarding quality of care, access to medicines, patient follow-up and care coordination.

***Hypertension registry***

According to several informants, a major challenge to the management of hypertension and CV diseases is the lack of a registry or any form of metric to monitor time trends. In 2000, there was a federal program called HyperDia (hypertension and diabetes) and, for the first time, a highly promising national registry of diabetes and hypertension was built to fill this gap. However, after two or three years, the registry was discontinued which was considered a big loss by organizations involved in NCD programmes. The reason for the discontinued was cited to be a change in government.

**NCD policy stream Brazil**

***Health system***

The creation of the Brazilian Unified Healthcare System (Sistema Único de Saúde; SUS) in 1989 was seen by some informants, as the single biggest achievement in terms of public policy in general.

The SUS is one of the best-known Universal Health Coverage models in Central- and South-America striving to provide access to healthcare to the entire population. More than 75% of the country’s population depend exclusively on SUS for health care.^38^ The SUS, comprised of both public and private health care institutions and providers, is responsible for most primary care services that are financed through contributions from the federal, state and municipal budgets. Brazil put in place a strong policy framework to tackle NCDs, including hypertension, and significant efforts were made to strengthen the capacities and coverage of primary healthcare under the SUS. According to several informants, the healthcare institution has proven resilient to political change in the country over the last 30 years.

***National NCD strategies & programs***

The first guideline and the first set of goals that the MoH has established around NCDs as a collective problem was the 10-year Strategic Action Plan to Tackle NCDs 2011–2022 aiming to develop and implement public policies for the prevention and control of major NCDs.^45^ The plan served to guide the actions of the countries’ states and municipalities to addressing NCDs, including hypertension, and four key modifiable risk factors – tobacco use, diet, physical inactivity, harmful use of alcohol – by leveraging the SUS.^46^ In 2020, the Minister opened a public consultation around the goals for the next 10-year cycle. Informants noted that, around the same time as the first National NCD Action Plan was being established, the ‘Vigitel’ survey was launched for the first time. The Vigitel, a national telephone survey covering the NCD risk factors and conducted across capitals every two years since 2006, was named to be the most widely cited and disseminated data reference in the country (see also Annex Problems stream).^47^ While there is a plethora of initiatives, programs, projects and surveys on sub-national level that may be relevant to NCD management and prevention, it was beyond this study to conduct a full review of all relevant activities.

***Tobacco***

Brazil started putting in place tobacco control laws in the late 1989s and has become a widely cited international reference for best practice.^48^ The country has managed to reduce the prevalence of smokers in the population from 35% in 1989 to 15% in 2021.^49-51^ It has done so by cigarette tax and price increases, plain packaging and smoke-free bans. Initial smoking restrictions were supported and strengthened by city, state and federal laws, resulting in the National Tobacco Control Policy adopted in 1986. In 1999, a tobacco regulatory body – the National Health Surveillance Agency (Agência Nacional de Vigilância Sanitária; ANVISA) was created rendering tobacco product regulation an important element of the Brazilian policy. The function is to controlling content, packaging and registration. Above all, ANVISA is responsible for periodic adjustments of the tobacco tax and the retail sales price of cigarettes. In 2011, the tax on industrialized goods and a minimum price for selling cigarettes were updated. In addition, smoking became prohibited in most private and public enclosed spaces ^52 53^ and health warnings of smoking on advertising and cigarette packs from 1988 have been reformulated and complemented with images. The most important pro-tobacco regulation actors seem to be i) government agencies; ii) the civil society: scientific and professional associations, NGOs, healthcare professionals, some farmers and merchants; and iii) part of the legislative and judiciary. Among the government agencies, a major role was attributed to the National Cancer Institute (Instituto Nacional de Cancer; INCA) – an MoH body responsible for the development and coordination of integrated actions in the prevention and control of cancer in Brazil.

***Nutritional policies***

Since 2007 Brazil’s Ministry of Health has worked with the Brazilian Association of Food Industries (Associação Brasileira da Indústria de Alimentos; ABIA), who make over 70% of all processed food in the country, to improve the nutritional profile of the food. This work initially focused on reducing trans-fatty acids has since led to setting regional salt targets and food reformulation. Health and nutrition have been incorporated into school curriculums and the Ministry of Health runs regular healthy eating campaigns, with salt reduction as a central focus. Strict standards are also in place for food served in cafeterias, both in schools and workplaces.^46^ The strategic promotion of a healthy diet to prevent NCDs is carried out by the MoHs National Policy on Food and Nutrition (Política Nacional de Alimentação e Nutrição – PNAN). In line with international standards, the PNAN establishes guidelines for the promotion of healthy food practices. One of the first policies in that domain – mandatory food labelling – has been adopted in 2003 in the framework of a harmonization process of Mercosur to take effect in 2007.^34^ The same year, saw an agreement between ABIA and the MoH to reduce the salt, sugar and trans-fat content in processed foods which was renewed in 2010. This policy update pertaining to processed foods has been included in the ‘National Action Plan for NCDs (2011–2022)’^45^ as well as in ‘The Ministry of Health’s Strategic Plan of Action for 2011–2015’.^54^

The issue of excess sodium in industrialized foods has been discussed by a suite of stakeholders, including MoH and ANVISA, professional associations (e.g. Brazilian Society of Cardiology, Brazilian Association of Nutrition), ABIA and consumer protection organizations resulting, in 2010, in a recommendation to reduce from currently 4700 mg to less than 2000 mg/day by 2020 in accordance with the PACO guidelines. This was followed up by an agreement signed in 2011 to consider establishing goals for the reduction of sodium in foods as priority.^55^

**NCD politics stream Brazil**

***Sustainability of NCD policies and programmes***

According to several informants, changes in the government have negatively affected NCD programmes and their continuation in the past across Brazil – although no more than in other countries with a 4–5 year political cycle and corresponding change of staff and agenda. Yet, the abortion of the federal program HyperDia (see Problem section in main article), that established the first national registry for hypertension and diabetes in 2000, was seen as a major blow to the NCD community at the time. On the other hand, the creation of the SUS – Brazil's publicly funded health care system – has led to a structure that has proven resilient to political change over decades owing to a wide political consensus surrounding it. After Brazil’s military dictatorship from the 1960s to the 1980s, the national Constitution sought to give the population more freedom and rights. Among the social development objectives set, improving health care was a priority according to informants. Created in 1989, SUS is one of the largest public health care system in the world, by number of beneficiaries and treatment centres and land area coverage. According to informants, the healthcare system now seems to be a driver for NCD action by delivering data and conducting NCD programmes and is seen as the single most important public policy in Brazil at all.

***Multilateral activities***

Brazil has demonstrated an exceptional political will for tobacco control since the late 1980s and has become a widely cited international reference for best practice. Having implemented cigarette tax and price increases, plain packaging and smoke-free bans already, Brazil was consulted early on in the negotiations of the Framework Convention on Tobacco Control (FCTC) that held the first international public health convention in 2003 under the auspices of the WHO.^56^ Consequently, occupied some key positions in the movement. The country acted as Vice-President of the Working Group that prepared the first draft of the convention, chaired the FCTC Intergovernmental Negotiation Body and headed the working group that prepared the first Conference of Parties.^57^ Brazil consolidated its position as an international leader for tobacco control by updating its tobacco policies regarding in 2011 and was appointed to preside the FCTC Secretariat in 2014.

Regarding nutritional policies, Brazil seemed update and quick to align with global and regional movements and the guidance from the Pan American Health Organization (PAHO) and WHO. For instance, food labelling made mandatory in Brazil in 2003^34^ in a harmonization process within Mercosur. This was aligned with the WHO and the Food and Agriculture Organization (FAO) recommendation in the same year, that ‘trans’ fat consumption should be less than 1% of the total daily energy calories as published in a report on Diet, Nutrition, and Chronic Disease Prevention.^58^

***Role of civil society***

There was a consent among the informants that the civil society in Brazil had an important role of in provided the scientific data and an understanding for the context and problem. Moreover, they identify and assemble the appropriate actors, above all, from the academic/medical sector and advocacy organizations. By doing so, they provide the scientific reasons for a given policy change. However, civil society members and bodies were seen as background actors rather than the ones at the front end, advocating for the policies themselves.

***Role of S*ã*o Paulo state***

Owing to the size and complexity of the Brazilian governance system (including 26 states and >5000 municipalities that are governed independently, it is – according to informants – quite frequent, that policies start at the municipal or state level in Brazil. Host to São Paulo city, being the largest and economically most important city in Central- and South America, São Paulo state/municipality has given rise to major national NCD policies. First, the fight for the full health care coverage for people with diabetes started in São Paulo state, according to sources. When this effort received approval in the early 2000s, it was scaled throughout the country, and finally implemented at the federal level. The same happened with smoking in public and private areas and closed places. All this all started in São Paulo State and started to be replicated for different states after 2009. Finally the policies were implemented in the whole country, 5–6 years after adoption in São Paulo.

**References**

1. Ministry of Health Mongolia. Mongolian STEPS survey on the prevalence of noncommunicable disease risk factors 2006. Ulaanbaatar, Mongolia: Ministry of Health Mongolia, World Health Organization, 2006.

2. Public Health Institute Mongolia and Kagawa NU, Japan Japan joint survey 2002, 2002.

3. Suvdaa Jea. Determination of prevalence of diabetes mellitus. Ulaanbaatar, 1999.

4. Nutrition Research Center of the Public health Institute. The Nutrition Status of the Mongolian Population 1999. The 2nd National Nutrition Survey, 2002. Ulaanbaatar, 2002.

5. Health; Mo. Mongolian STEPS survey on the prevalence of noncommunicable disease and injury risk factors-2009. Ulaanbaatar, Mongolia: Ministry of Health; World Health Organization, 2009.

6. Ministry of Health Mongolia, World Health Organization. Third national STEPS survey on the prevalence of noncommunicable disease and injury risk factors-2013. Ulaanbaatar, Mongolia, 2013.

7. Enkhtungalag B, Batjargal J, Chimedsuren O, et al. Developing a national salt reduction strategy for Mongolia. *Cardiovasc Diagn The* 2015;5(3):229-37. doi: 10.3978/j.issn.2223-3652.2015.04.11

8. Sheiman I, Fleck F. Rocky road from the Semashko to a new health model. *B World Health Organ* 2013;91(5):320-21. doi: 10.2471/Blt.13.030513

9. Dugee O, Munaa E, Sakhiya A, et al. Mongolia's Public Spending On Noncommunicable Diseases Is Similar To The Spending Of Higher-Income Countries. *Health Affair* 2017;36(5):918-25. doi: 10.1377/hlthaff.2016.0711

10. Chimeddamba O, Peeters A, Walls HL, et al. Noncommunicable disease prevention and control in Mongolia: a policy analysis. *Bmc Public Health* 2015;15 doi: ARTN 66010.1186/s12889-015-2040-7

11. Ministry of Health Mongolia. National programme on prevention and control of noncommunicable disease. Government resolution no 246. Ulaanbaatar: Government of Mongolia, 2007.

12. WHO Framework Convention on Tobacco Control. Mongolia – comprehensive tobacco control legislation revised 2013 [Available from: <https://www.who.int/fctc/implementation/news/news_mon/en/> accessed 19.10.2020.

13. UN Interagency Task Force on NCDs. Prevention and control of noncommunicable diseases in Mongolia - the case for investment, 2017.

14. Republic du Senegal. Enquete nationale sure les facteurs de risque des maladies non transmissibles STEPS2015 - rapport préliminaire: les indicateurs‐clés: World Health Organization, 2016.

15. World Health Organization. Noncommunicable diseases country profiles 2018: WHO, 2018.

16. World Bank. The World Bank in Senegal 2020 [Available from: <https://www.worldbank.org/en/country/senegal/overview#1> accessed 10.02.2021.

17. World Health Organization. Global Health Expenditure Database 2017 [Available from: <https://apps.who.int/nha/database> accessed 10.02.2021.

18. World Health Organization. WHO country cooperation strategy at a glance: Senegal G [Available from: <https://apps.who.int/iris/handle/10665/136843> accessed 10.02.2021.

19. Agence Nationale de la Statistique et de la Démographie. Enquete continue sur la prestation des services de soins de sante (ECPSS) - Senegal 2015. Senegal: Ministère de la Santé et de l’Action Sociale, 2015.

20. World Health Organization. Essential Medicines and Health Products Information Portal 2020 [Available from: <http://apps.who.int/medicinedocs/documents/s22103en/s22103en.pdf> accessed 27.10.2021.

21. Ministere de la Sante et de la Prevention. Plan national de developpement sanitaire PNDS 2009-2018. Republique du Senegal: Ministere de la Sante et de la Prevention, 2009.

22. World Health Organization. Senegal NCD country profile 2014. In: Organization WH, ed., 2014.

23. Lachat C, Nago E, Ka A, et al. Landscape Analysis of Nutrition-sensitive Agriculture Policy Development in Senegal. *Food Nutr Bull* 2015;36(2):154-66. doi: 10.1177/0379572115587273

24. Ministere de la Sante et de l'Action Sociale. Lutte contre les maladies non transmissibles 2017-2020. Plan Strategique. Dakar, Senegal, 2017.

25. Ministere de la Sante et de l'Action Sociale. Plan national de développement sanitaire et social (PNDSS) 2019-2028. République de Sénégal: Ministere de la Sante et de l'Action Sociale, 2019.

26. World Health Organization. WHO Report on the global tobacco epidemic, 2017 - Monitoring tobacco use and prevention policies. Geneva: WHO, 2017.

27. World Health Organization. WHO Report on the global tobacco epidemic, 2019 - Offer help to quit tobacco use. Geneva: WHO, 2019.

28. World Health Organization. Global alcohol report - Senegal Country Profile In: WHO, ed. Geneva, 2018.

29. The East African. Senegal to offer free breast and cervical cancer treatment 2019 [Available from: <https://www.theeastafrican.co.ke/tea/science-health/senegal-to-offer-free-breast-and-cervical-cancer-treatment-1427446> accessed 09.02.2021.

30. World Health Organization. World Cancer Report - Senegal Country Profile 2020. In: WHO, ed. Geneva, 2020.

31. World Health Organization. Diabetes country profiles, 2016 - Senegal. In: WHO, ed. Geneva, 2016.

32. Jardim PCBV, de Souza WKSB, Lopes RD, et al. I RBH - first Brazilian hypertension registry. *Arq Bras Cardiol* 2016;107(2):93-98. doi: 10.5935/abc.20160120

33. Mengue SS, Bertoldi AD, Ramos LR, et al. Access to and use of high blood pressure medications in Brazil. *Rev Saude Publ* 2016;50 doi: ARTN 8s10.1590/S1518-8787.2016050006154

34. Block JM, Arisseto-Bragotto AP, Feltes MMC. Current policies in Brazil for ensuring nutritional quality. *Food Qual Saf-Oxford* 2017;1(4):275-88. doi: 10.1093/fqsafe/fyx026

35. Ministry of Health. VIGITEL Brazil 2017. Brasilia, DF, 2016.

36. Brazilian Institute of Geography and Statistics. Pesquisa de orcamentos familiares 2008-2009 Brazilian Institute of Geography and Statistics, 2011.

37. Fernandes JG. Stroke prevention and control in Brazil: missed opportunities. *Arq Neuro-Psiquiat* 2015;73(9):733-35. doi: 10.1590/0004-282x20150127

38. Brazil's march towards universal coverage. *B World Health Organ* 2010;88(9):646-47. doi: 10.2471/Blt.10.020910

39. Macinko J, Harris MJ. INTERNATIONAL HEALTH CARE SYSTEMS Brazil's Family Health Strategy - Delivering Community-Based Primary Care in a Universal Health System. *New Engl J Med* 2015;372(23):2177-81. doi: 10.1056/NEJMp1501140

40. Presidência da República Casa Civil SpAJ. Lei Nº 13.414, de 10 de Janeiro de 2017, 2017.

41. brazzil. Brazil cuts 5 million people from its popular social program Bolsa Família 2018 [Available from: <http://brazzil.com/brazil-cuts-5-million-people-from-its-popular-social-program-bolsa-familia/> accessed 02.03.2021.

42. Pierin AMG, Marroni SN, Taveira LAF, et al. Hypertension control and related factors at primary care located in the west side of the city of Sao Paulo, Brazil. *Cienc Saude Coletiva* 2011;16:1389-400. doi: Doi 10.1590/S1413-81232011000700074

43. Mion D, Pierin AMG, Bensenor IM, et al. Hypertension in the City of Sao Paulo: Self-Reported Prevalence Assessed by Telephone Surveys. *Arq Bras Cardiol* 2010;95(1):99-105. doi: Doi 10.1590/S0066-782x2010005000051

44. Campos CL, Pierin AMG, Pinho NA. Hypertension in patients admitted to clinical units at university hospital: post-discharge evaluation rated by telephone. *Einstein (Sao Paulo)* 2017;15(1):45-49. doi: 10.1590/S1679-45082017AO3862 [published Online First: 2017/04/27]

45. Ministry of Health Brazil. Strategic action plan to tackle noncommunicable diseases (NCD) in Brazil 2011-2022. Brasilia-DF, Brasil: Ministry of Health Brazil, 2011.

46. Schmidt MI, Duncan BB, Silva GAE, et al. Health in Brazil 4 Chronic non-communicable diseases in Brazil: burden and current challenges. *Lancet* 2011;377(9781):1949-61. doi: 10.1016/S0140-6736(11)60135-9

47. Bernal RTI, Malta DC, Claro RM, et al. Effect of the inclusion of mobile phone interviews to Vigitel. *Rev Saude Publ* 2017;51 doi: ARTN 15s

10.1590/S1518-8787.2017051000171

48. Romero LC, da Costa e Silva VL. 23 anos de controle do tabaco no Brasil: a atualidade do programa nacional de combate ao fumo de 1988. *Revista Brasilea de Cancerologia* 2011;57(3):305-14.

49. Malta DC, Oliveira TP, Luz M, et al. Smoking trend indicators in Brazilian capitals, 2006-2013. *Cienc Saude Coletiva* 2015;20(3):631-40. doi: 10.1590/1413-81232015203.15232014

50. Monteiro CA, Cavalcante TM, Moura EC, et al. Population-based evidence of a strong decline in the prevalence of smokers in Brazil (1989-2003). *B World Health Organ* 2007;85(7):527-34. doi: 10.2471/Blt.06.039073

51. Levy D, de Almeida LM, Szklo A. The Brazil SimSmoke policy simulation model: the effect of strong tobacco control policies on smoking prevalence and smoking-attributable deaths in a middle income nation. *Plos Med* 2012;9(11) doi: ARTN e100133610.1371/journal.pmed.1001336

52. Presidência da República Casa Civil SpAJ. Decreto Nº 8.262, de 31 de maio de 2014 2014 [Available from: <http://www.planalto.gov.br/ccivil_03/_ato2011-2014/2014/decreto/d8262.htm> accessed 02.03.2021.

53. Presidência da República Casa Civil SpAJ. Lei Nº 12.546, de 14 de decembro de 2011 2011 [Available from: <http://www.planalto.gov.br/ccivil_03/_ato2011-2014/2011/lei/l12546.htm> accessed 02.03.2021.

54. Ministry of Health. Planejamento Estratégico do Ministério da Saúde 2011 – 2015 - Results and Perspectives: Ministry of Health Brasilia - DF, 2013.

55. Ministry of Health. Termo de Compromisso N°004/2011: Ministry of Health, Brazilian Association of Food Industries (ABIA); Brazilian Association of food Pasta Industries ABIMA; the Brazilian

Wheat Industry Association (ABITRIGO); Brazilian Bakery and Confectionery Association (ABIP), 2011.

56. World Health Organization. WHO Framework Convention on Tobacco Control: WHO,, 2003.

57. Saldanha PM. Convencao do tobaco da OMS - Gênese e papel da presidência brasileira nas negociações. Brasilia, 2015.

58. World Health Organization. Diet, nutrition and the prevention of chronic diseases. WHO Technical Report Series, No 916 (TRS 916): WHO, 2003.
